# Open source software toolchain for automated non‐targeted screening for toxins in alternative foods

**DOI:** 10.1016/j.mex.2021.101551

**Published:** 2021-10-14

**Authors:** S.W. Breuer, L. Toppen, S.K. Schum, J.M. Pearce

**Affiliations:** a Department of Electrical and Computer Engineering, Michigan Technological University, United States; b Department of Environmental Engineering, Michigan Technological University, United States; c ChARM lab, Michigan Technological University, United States; d Department of Electrical and Computer Engineering, Western University, Canada

**Keywords:** Alternative food, Edible leaves, Edible plants, Existential risk, Global catastrophic risk, Leaf concentrate, Leaf protein, Non-target screening, Sustainable food systems, Toxins

## Abstract

Previous published methods for non-targeted screening of toxins in alternative foods such as leaf concentrate, agricultural residues or plastic fed to biological consortia are time consuming and expensive and thus present accessibility, as well as, time-constraint issues for scientists from under resourced settings to identify safe alternative foods. The novel methodology presented here, utilizes a completely free and open source software toolchain for automatically screening unknown alternative foods for toxicity using experimental data from ultra-high-pressure liquid chromatography and mass spectrometry. The process uses three distinct tools (mass spectrometry analysis with MZmine 2, formula assignment with MFAssignR, and data filtering with ToxAssign) enabling it to be modular and easily upgradable in the future. MZmine 2 and MFAssignR have been previously described, while ToxAssign was developed here to match the formulas output by formula assignment to potentially toxic compounds in a local table, then look up toxic data on the Open Food Tox Database for the matched compounds. This process is designed to fill the gap between food safety analysis techniques and developing alternative food production techniques to allow for new methods of food production to be preliminarily tested before animal testing. The methodology was validated against a previous method using proprietary commercial software. The new process identifies all of the toxic elements the previous process identified with more detailed information than the previous process was able to provide automatically.

• Efficient analysis to find potentially toxic compounds in alternative foods and resilient foods.
• Identification of potentially unsafe products without the use of live animal testing.
• Modular free and open source design to allow for upgrading or fitting of user needs.

## Graphical abstract

**Figure:**
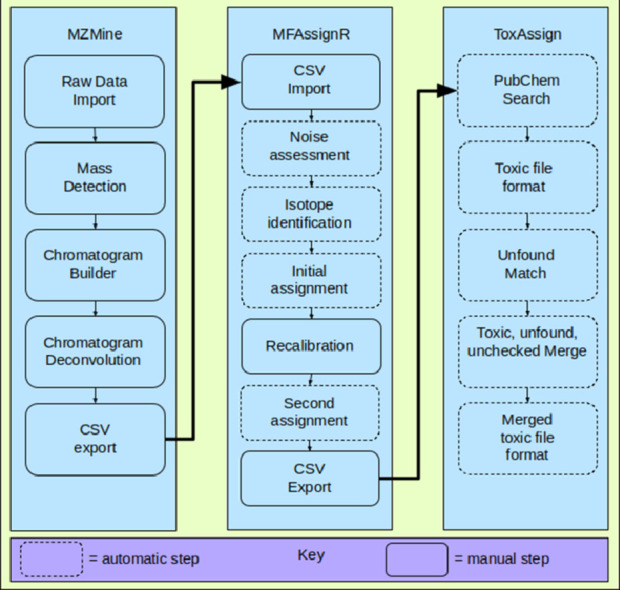

Specifications table

**Table:** 

Subject Area:	Pharmacology, Toxicology and Pharmaceutical Science Pharmacology, Toxicology and Pharmaceutical Science
More specific subject area:	*Toxic screening*
Method name:	*Open Source Automated Non-Targeted Screening for Toxins in Alternative Foods*
Name and reference of original method:	Pearce, J.M., Khaksari, M. and Denkenberger, D., 2019. Preliminary automated determination of edibility of alternative foods: Non-targeted screening for toxins in red maple leaf concentrate. *Plants, 8*(5), 110; https://doi.org/10.3390/plants8050110.
Resource availability:	https://osf.io/nh76z/

## Method details

 

## Introduction

Agricultural-loss-based global catastrophic risks (GCRs) have some of the greatest probabilities and impacts [1]. Previous work has shown alternative food supplies made from converting fossil fuels, wood and leaves to human-edible food could feed humanity even in the event of a GCR that eliminated all conventional agriculture [2], [3], [4]. Using a variety of alternative foods can provide a balanced diet of macronutrients [5] and micronutrients [6] to maintain human life. It is also cost effective to prepare for alternative food production both globally [6] and in the U.S. [7]. For alternative foods to be effective in GCRs they must be to maintain caloric intake consistently. This can be a challenge in the event of a sun-blocking GCR because there is a gap between the time that stored foods run out globally (∼6 months) and the to ramp up production of alternative foods that do not rely on sunlight (∼12 months) [2]. To date the best theoretical solution for this transition problem is to use leaves killed by the GCR [8]. It is possible to grind and press the leaves, and then coagulating the resultant liquid as leaf concentrate, which contains ∼8% of the dry matter of the original leaves while the remaining liquid contains much of the toxins and is discarded [9,10]. Although technically, viable there is an enormous knowledge gap on the toxicity of leaf concentrate for humans from the most common tree leaves. Traditionally, the approach for detection of toxic compounds in a solution is to do targeted screening by purchasing the corresponding reference standards for identification [11]. This method is unrealistic for the potential toxic compounds in plant materials as there are over 1500 phytotoxins already identified in the Toxic Plants–Phytotoxin (TPPT) Database [12]. Fortunately, high-resolution mass spectrometry (HRMS) has allowed for non-targeted screenings where no prior information is available for identification of unknowns in a sample [14]. Further, a recent study, provided the preliminary steps for obtaining a rapid toxics screening process of common leaf concentrates to be used for alternative foods [13]. In non-targeted screening, experimental evidence is needed to confirm the identification using a suitable algorithm. This evidence includes accurate mass, isotope pattern, presence of additional adducts, retention time, fragmentation information, and other experimental evidence. The quantity and quality of evidence available for identification leads to a range of levels of confidence for compound identification [11,^2^,15]. Pearce et al.’s non-targeted approach used an ultra-high-resolution hybrid ion trap orbitrap mass spectrometer (MS) coupled to an ultra-high-pressure two-dimensional liquid chromatograph (LC) system on the most common leaf in North America, red maple (acer rubrum) [16], to provide the greatest potential for alternative food [13]. The data was analyzed using the ThermoFisher Scientific's screening method of unwanted compounds in food [17] and relied on a proprietary software package - the Thermo Scientific Compound Discoverer software [18] for identification of unknown compounds. This software costs $20,000 per seat [18]. Then the identified chemicals were cross-referenced pseudo-manually among several databases to identify toxic and harmful chemicals. Although this process was effective, it was time consuming and expensive and thus presented both accessibility issues for scientists from under resourced countries to prepare their own lists of safe leaf concentrates for alternative foods as well as time barriers to screen all potential leaf sources and other alternative foods (e.g. agricultural residues or plastic fed to biological consortia). To overcome these limitations this methodology utilizes a completely free and open source software toolchain for automatically screening the experimental data from unknown alternative foods for toxicity.

## Experimental

Leaf concentrate is prepared following standard procedures [9,10]. For LC/MS analysis, leaf concentrate was diluted 12 times in water–acetonitrile 80:20 (v:v) and filtered with a 0.2 µm quartz filter. A Thermo Scientific Dionex Ultimate 3000 standard system was then used as a high-pressure liquid chromatography (HPLC) system on the material. The instrument was calibrated externally with Thermo Pierce calibration solution before LC/MS runs. Following [13] the analytical column was Phenomenex reversed-phase Kinetex XB-C18, 150 × 2.1 mm, 100 A˙, with 1.7 µm particle size. Mobile phase A was 0.1% formic acid in 100% LC/MS grade water and mobile phase B was 0.1% formic acid in LC/MS grade acetonitrile–water 95:5 (v:v) solution. Using a constant flow rate of 0.2 mL/min (0.2 mL/min was used due to small particle size of the column (1.7 um), which increases the back pressure), the mobile phase gradient was: 0 min; 5%B, 5 min; 5%B, 65 min; 90%B, 70 min; 90%B. The column was equilibrated with mobile A for 15 min before the next injection. The column oven was set at 35°C, and the full loop injection volume was set at 5 µL. The mass spectrometry instrument was a Thermo Scientific Orbitrap Elite equipped with electrospray ionization (ESI). The resolving power for accurate mass measurement during the LC/MS run was 120 K defined at m/z 400. The sample was run with both positive and negative ESI modes under two separate LC/MS runs. All the masses in the range of 100–600 m/z were recorded with full scan mode. In addition to the full scan, data-dependent MS/MS fragmentation was also recorded for the 5 tallest peaks on each spectral scan with a collision energy of 25 (arbitrary unit) to help identify co-eluting compounds.

## Data analysis

**Fig. 1:**
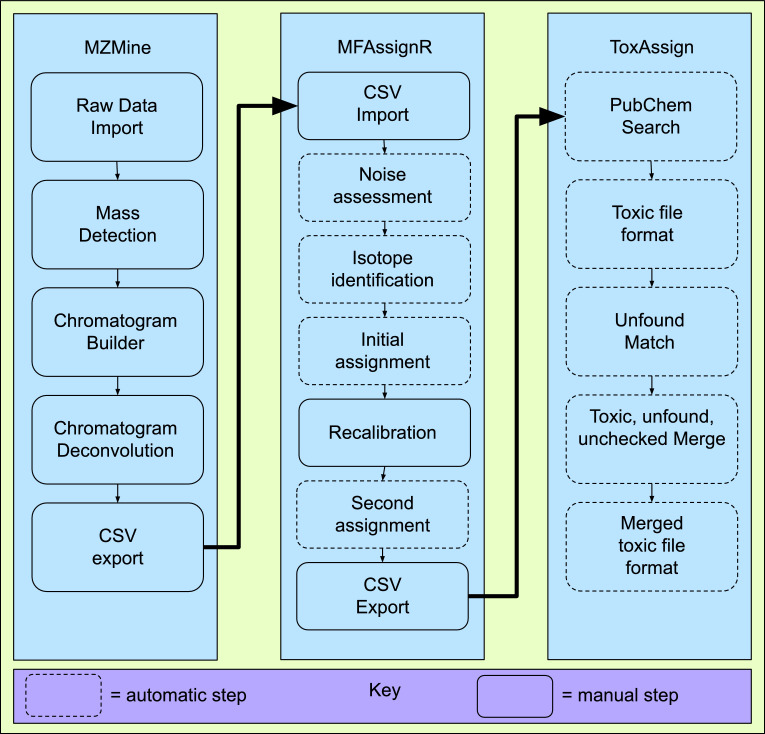
Map of full open source process for screening toxicity of unknown alternative foods.

This analysis method, which differs substantially from the original analysis by Pearce et al. as outlined in Fig. 1, consists of four parts, those being primary analysis by MZmine 2 [19,20], formula assignment by MFAssignR [21], filtering using PubChem [24] and a new open source python API caller known as ToxAssign [26], and final analysis by hand. Further confirmation of the toxic compounds identified can be performed using retention time and known pure samples following standard protocols [14].

### MZmine analysis

The first section of this analysis relies on the software, outlined in the paper by Pluskal et al. [20], MZmine to convert the RAW output files from the mass spectrometer. This software uses multiple steps to filter and identify mass peaks as well as retention time data, including mass detection and chromatogram-based analysis. Fig. 1 shows this analysis first uses the software to import the raw data, then detect the mass peaks in the data, use those mass peaks to build a chromatogram, then perform chromatogram deconvolution before outputting data to a CSV file. The chromatogram builder and deconvolution step are of particular note because of their ADAP, or automated data analysis pipeline, method outlined in the paper by Myers et al. [27].

*Raw Data Import:*

**Fig. 2:**
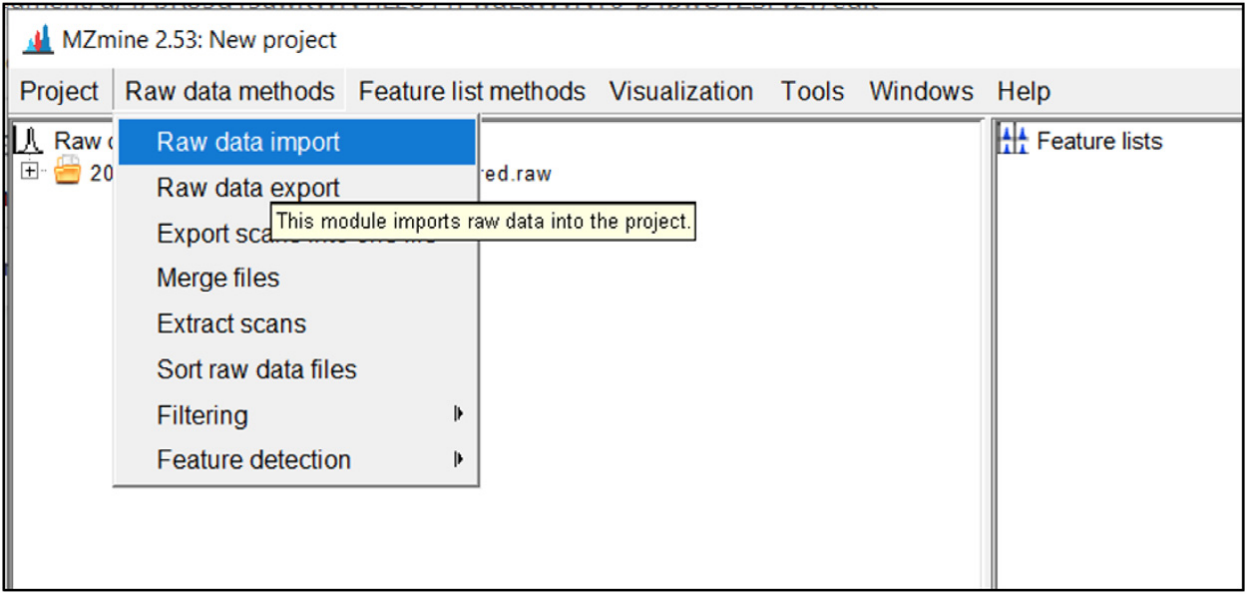
Screenshot to import raw data files.

To import a raw data file click on *Raw Data Methods → Raw Data Import* as shown in Fig. 2. This will open a window where the files may be selected from computer or hard drive storage. The imported files will then appear on the left-hand window with a label *Raw data files.*

**Fig. 3:**
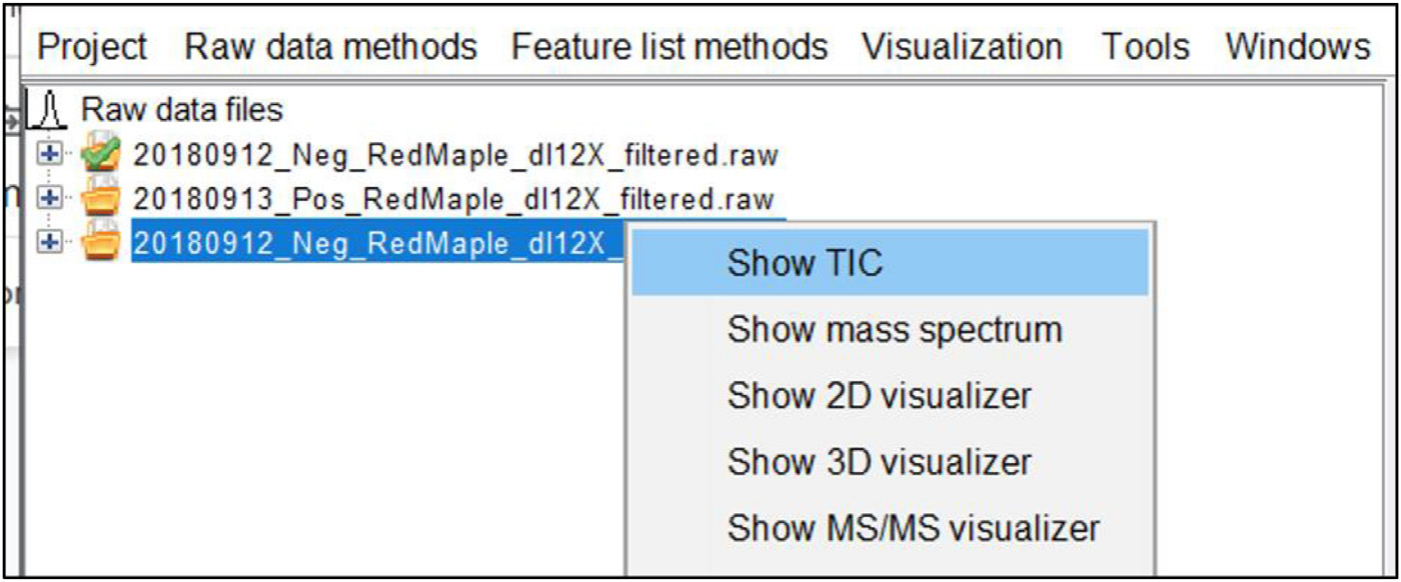
Screenshot to show initial chromatogram of raw data.

After the raw data is imported, it can be helpful to get an initial view of the chromatogram to determine the retention time where peaks are detected. To do so, hover the mouse over the desired raw data file on the left window and select show TIC on the dropdown menu as shown in Fig. 3.

**Fig. 4:**
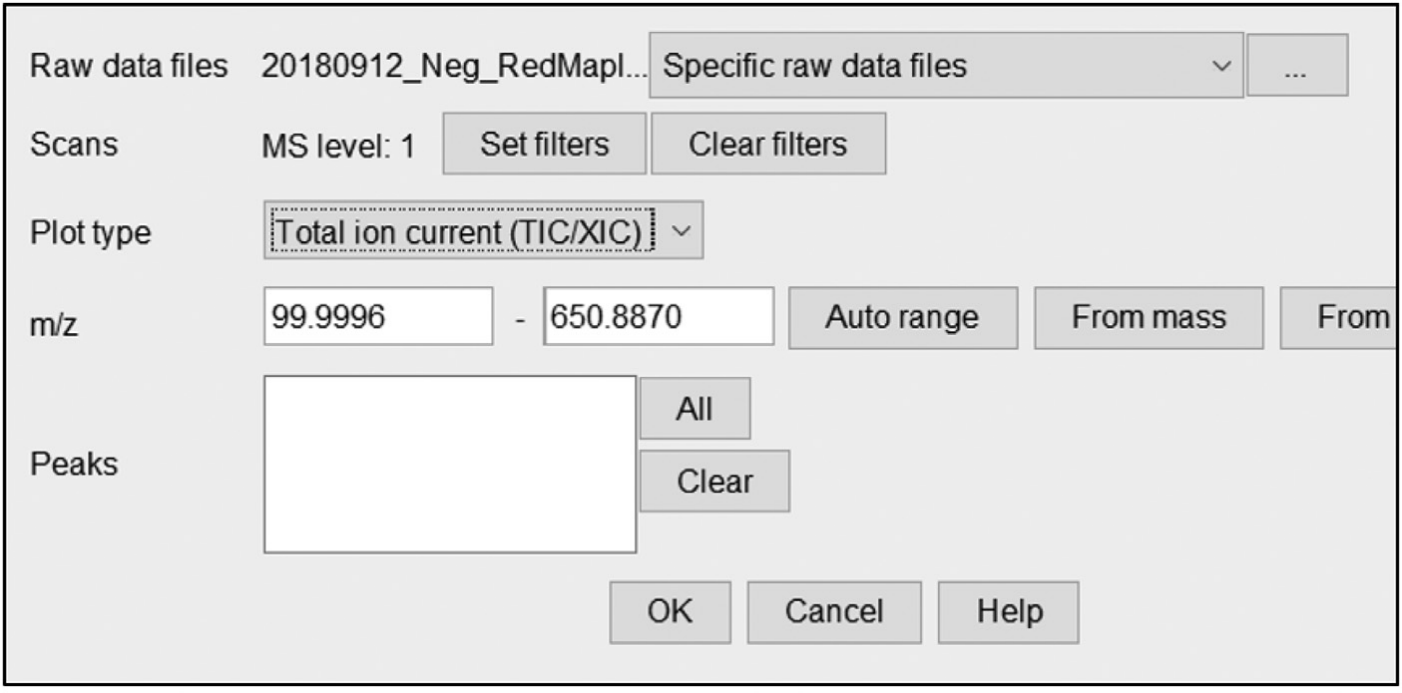
Parameter window to view initial chromatogram of raw data load.

This will bring up a parameter menu as shown in Fig. 3. Select Total ion current in the plot type dropdown menu and select Auto range for m/z, then click ok to display the chromatogram (Fig. 4).

**Fig. 5:**
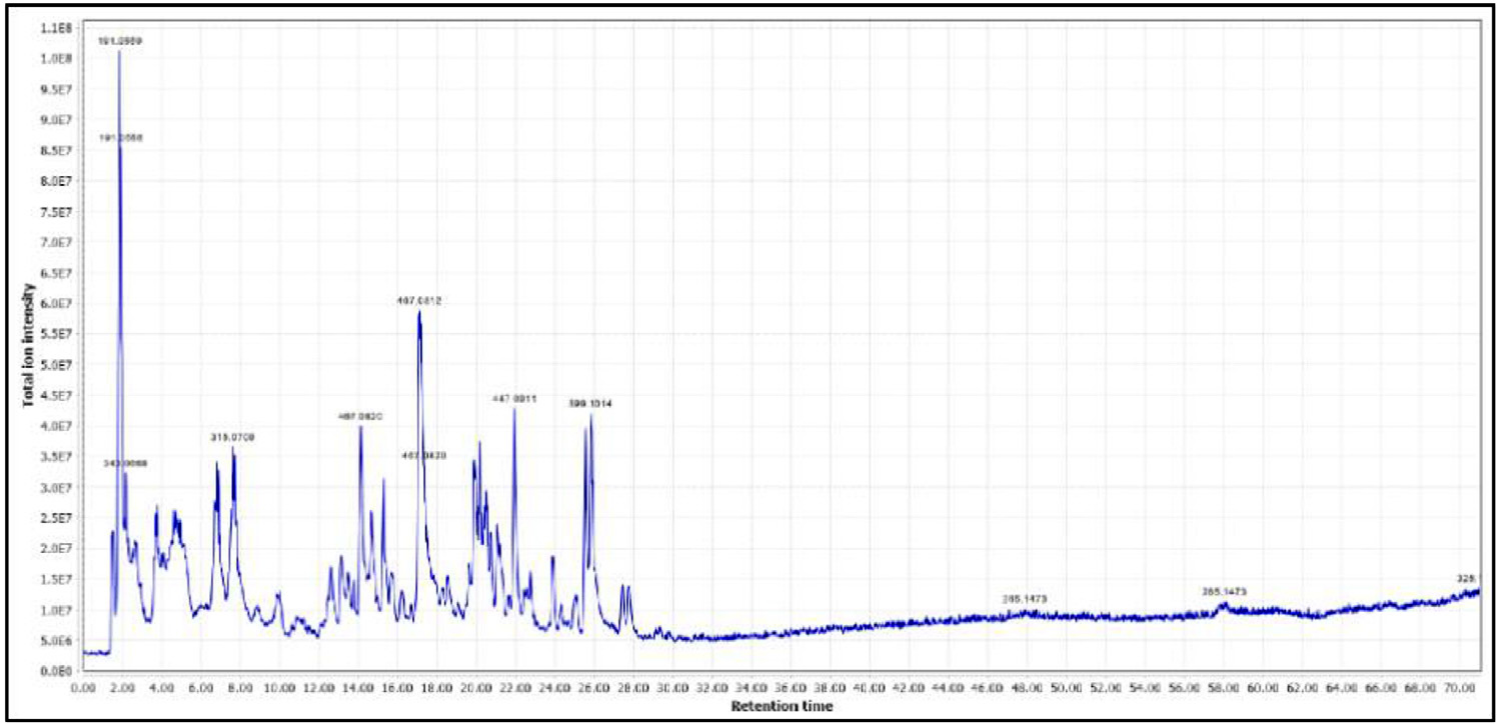
Example chromatogram of raw data to cut down retention time in future steps of analysis.

An example of an initial chromatogram is shown in Fig. 5, this is an example of a data set that can have its retention time cut down because of the clarity that there are no detected peaks after 30 min of retention time. Taking this extra step to shave down the amount of data to analyze can save a lot of time and better concentrate desired data.

*Mass detection:*

**Fig. 6:**
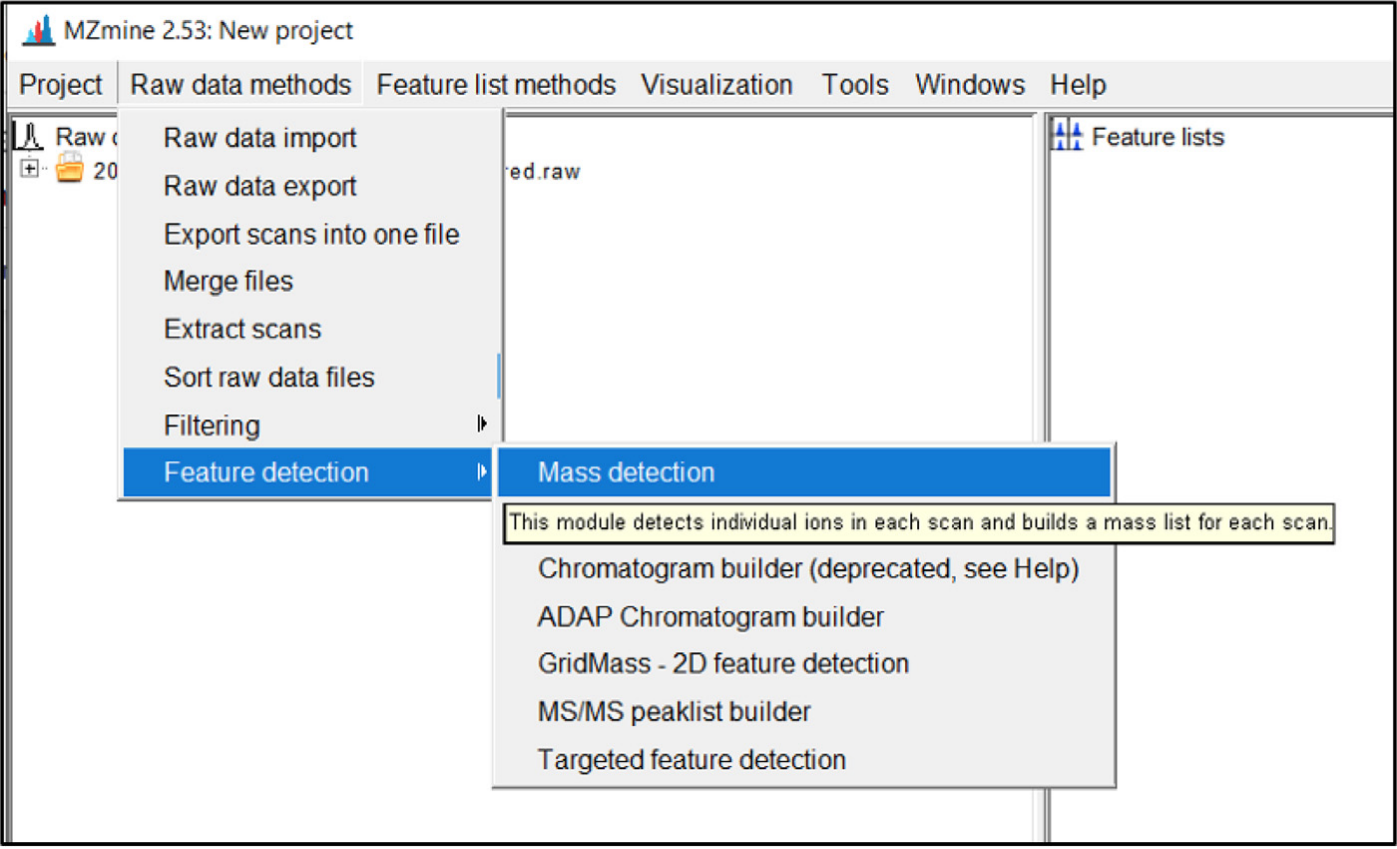
Screenshot to run mass detection.

The next step is to run the mass spectra through the peak detection feature to detect the masses from the data. Select the imported files by clicking on them in the left window, then click *Raw data methods → Peak detection → Mass detection* as shown in Fig. 6.

**Fig. 7:**
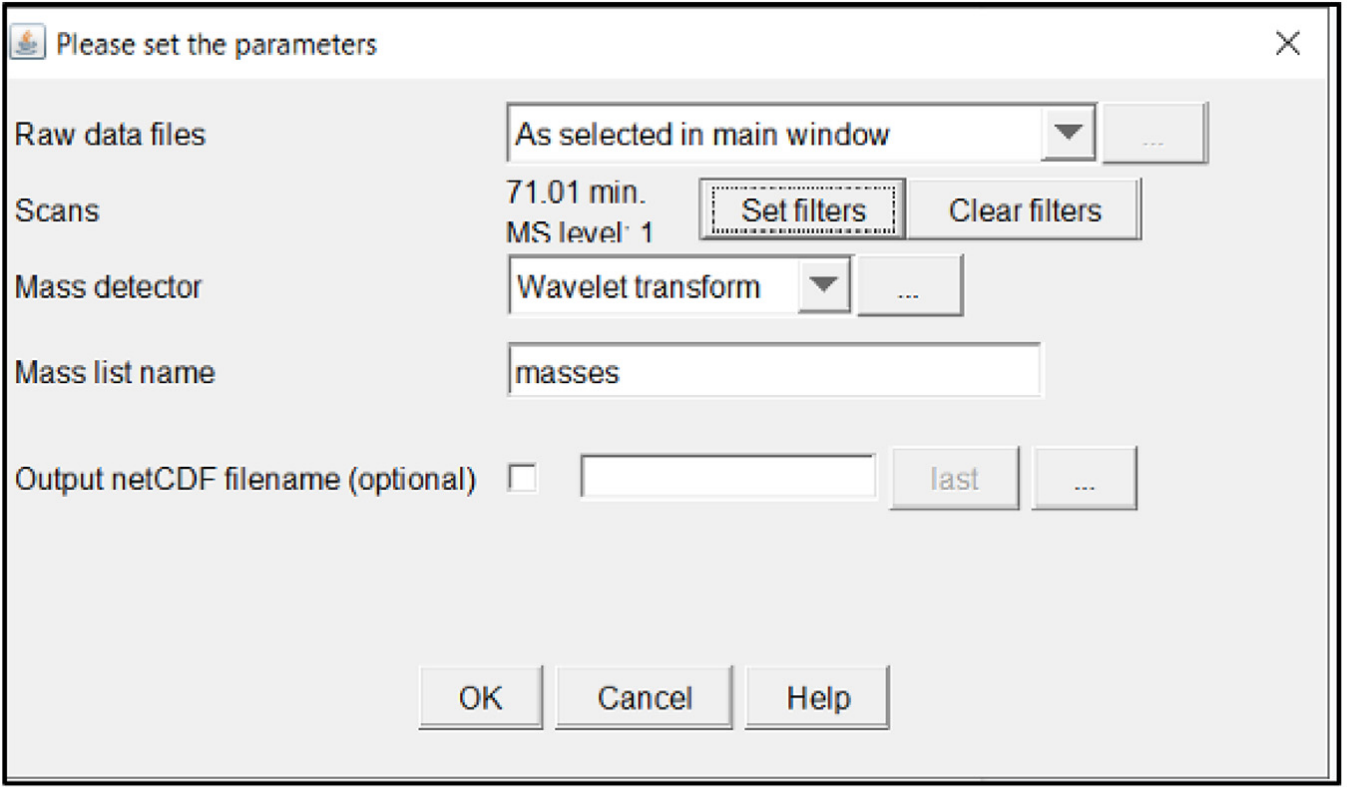
Mass detection parameter window.

This will open a window with several parameters to be entered. The next section will outline each parameter section shown on the right-hand side of Fig. 7.

*Mass detector:*

First step is to set the mass detector, an algorithm to use for mass detection and its parameters. Click the drop-down box titled *mass detector* and select *Exact Mass.*

**Fig. 8:**
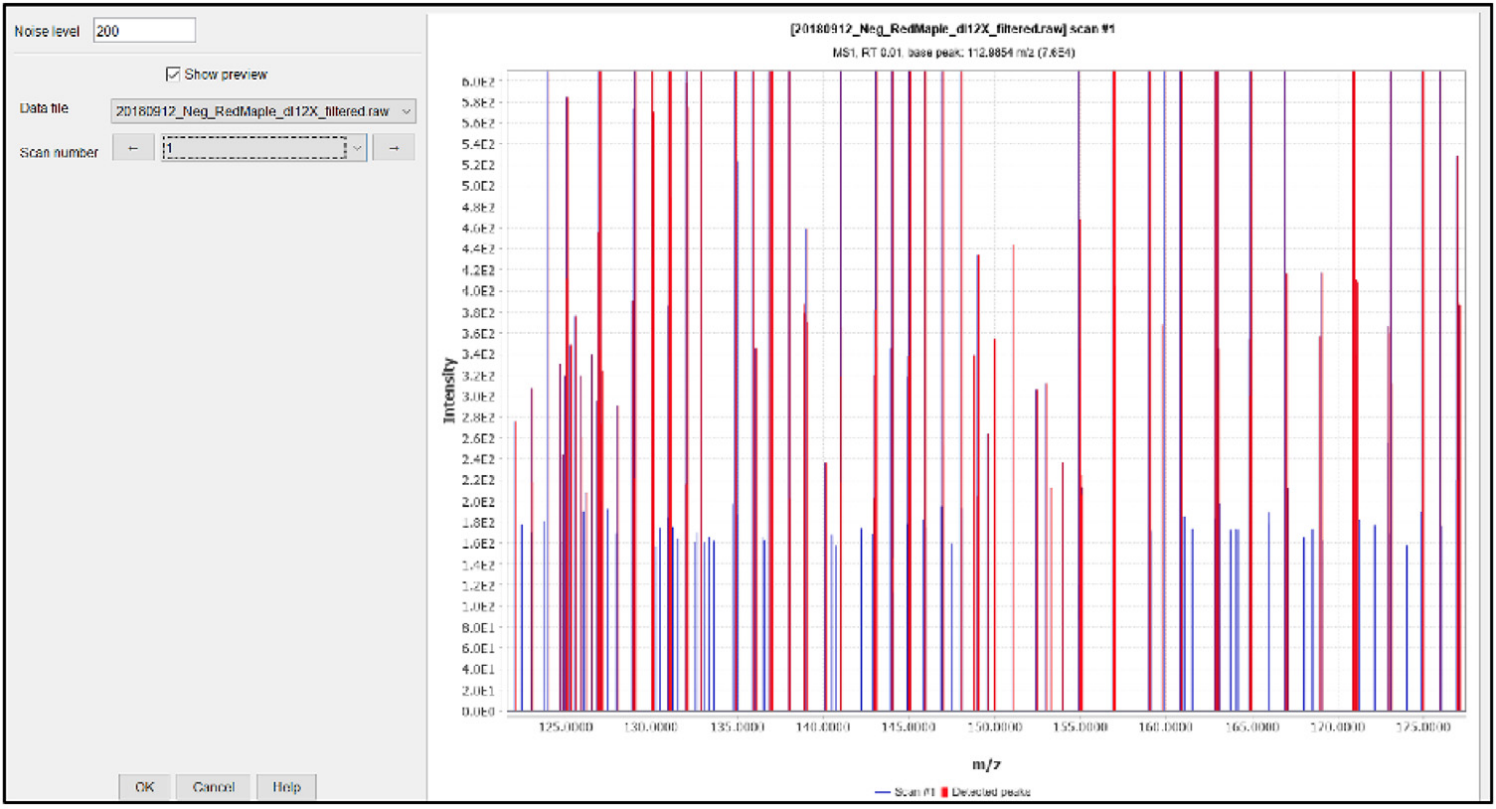
Setting and previewing noise level.

Then set the noise level to what matches the data, click the ellipses box to the right of the mass detector drop down box. This will open a parameter window where the noise can be set and previewed. The blue lines in Fig. 8 represent those that have not been selected and in red the ones that have. Previewing different noise levels can help determine what level is best to set the noise at. Noise should be roughly the same level across.

*Mass list name*:

The mass list name is what the name of the detected mass file will be outputted as after the feature is run it will generate a list of points for each scan in the sample. The program auto inputs *masses* as the title.

**Fig. 9:**
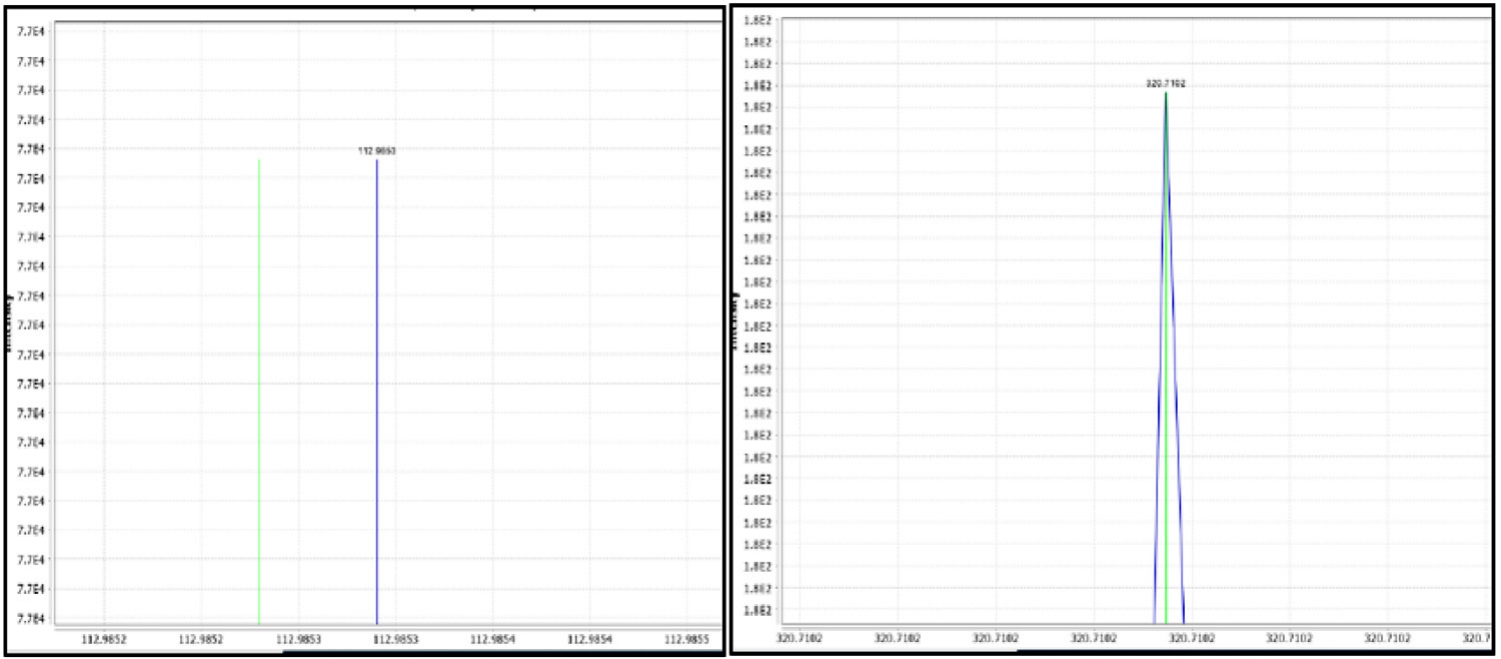
Checking mass detection feature with raw data.

Once all parameters are set appropriately for the data set, click the *OK* button to run the program. The program progress status is shown in the bottom right side of the window. When mass detection is complete a green check will appear over the file icon next to raw data on the left-hand side of the window. After the process is finished, click on the plus sign box to the left of each data file, this will display a list of the profile spectra. Then click on the plus sign box to the left of each profile spectrum and this will display the centroid spectrum labelled as the mass list name *masses.* Double click on the *masses* brings up a window that shows a profile spectrum in blue and the centroid masses in green. The accuracy of the mass detection step can be evaluated by comparing where the centroid spectrum and the profile spectrum line up. The goal is to have the green centroid line intersect the peak of the profile spectra in blue, or if there is no peak, for the green line to run closely parallel to the blue line. An example is shown in Fig. 9.

*ADAP chromatogram builder:*

The next step is to use the ADAP chromatogram builder feature, which builds extracted ion chromatograms by taking the mass lists and builds chromatograms for each mass that can be detected continuously over spectrometry scans.

**Fig. 10:**
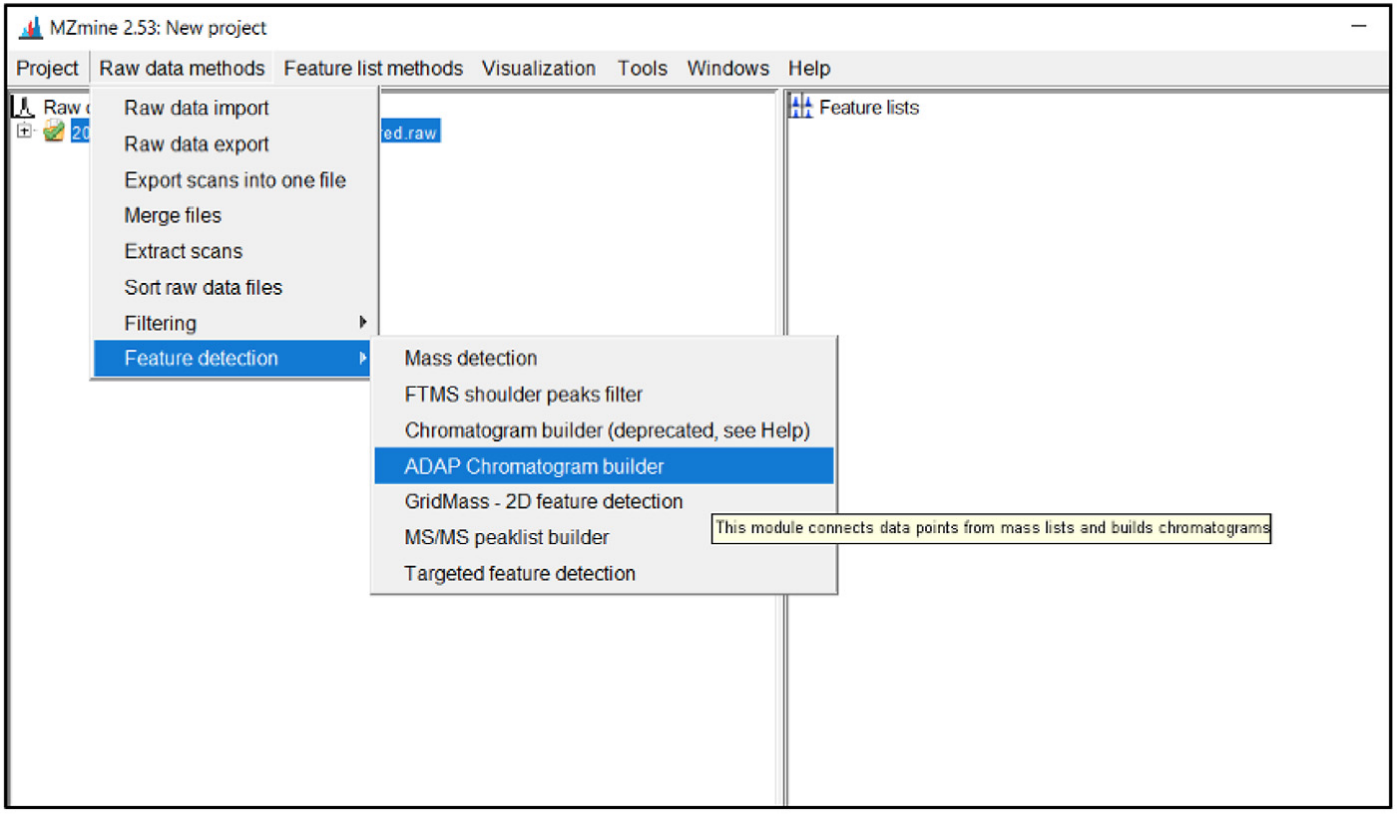
Screenshot to run ADAP chromatogram builder.

To run this feature, click *Raw data methods → peak detection → ADAP chromatogram builder* as shown in Fig. 10.

**Fig. 11:**
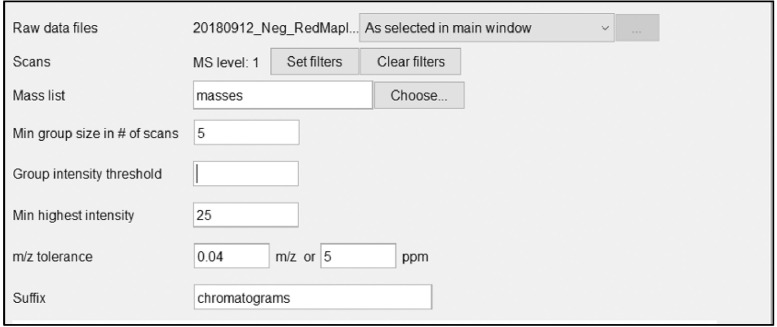
Example of ADAP chromatogram building parameter.

This will display a parameter window for the ADAP chromatogram building. The window of parameters is shown in Fig. 11 with descriptions of each parameter section following.

*Scans:*

Enter MS level 1 and the scan filters will be the same from what was set in the mass detection step of the process, scan parameters are not required unless needed to manipulate the raw data set.

*Mass list:*

Enter “masses” or whatever was set as the title for the mass detection results to be used for this program

*Min group size in # of scans:*

Minimum scan span over which some peak in the chromatogram must have (continuous) points above the noise level to be recognized as a chromatogram. The chromatography system will determine the optimal value. By evaluating the raw data and it is possible to observe the typical time span of chromatographic peaks and select appropriate values. Typically, a value of 3 to 5 will provide accurate results, 5 will work in most situations. The min group size number of scans detects peaks, so a setting at 3 will detect more peaks but with less confidence than a selection of 5. Selecting 3 can be helpful for small peaks that are eluted very quickly and not observed in many scans.

*Group intensity threshold:*

This parameter is the intensity value for which intensities greater than this value can contribute to the minimumScanSpan count. This value should be set to match the noise level (determined by analyzing the chromatogram in the mass detection step) exactly to keep the data set consistent as the chromatogram is being constructed.

*Min highest intensity:*

Points below this intensity will not be considered in starting a new chromatogram. The minimum highest intensity should be higher than the noise level. If 200 is entered for noise and intensity threshold, 300–400 would be a good minimum highest intensity, this can be raised to as high as 20,000 based on the minimum intensity used at Michigan Tech in past toxicology projects. Again, this can be adjusted based on results. This parameter should stay consistent between ESI negative and ESI positive datasets. It should be noted that the noise levels can be different between the two ionization modes sometimes, so this may not always be the best option.

*m/z tolerance:*

Maximum allowed difference between two m/z values to be considered the same chromatogram. The value is specified both as absolute tolerance (in m/z) and relative tolerance (in ppm). The tolerance range is calculated using maximum of the absolute and relative tolerances. If there is greater confidence in using either m/z or ppm, any value set to 0.0 will not be used. Typical values for m/z tolerance are 0.015 m/z and 3 ppm.

**Fig. 12:**
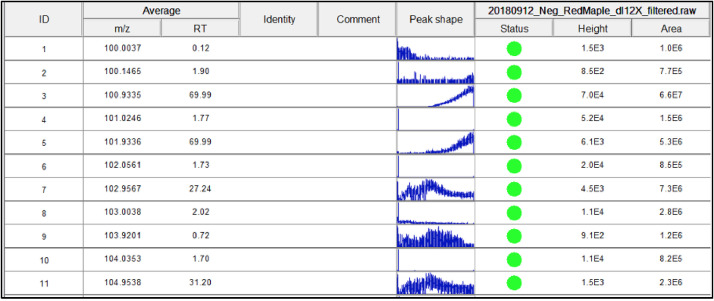
Screenshot of sample output from ADAP Chromatogram builder.

Fig. 12 shows a sample output results after running the ADAP Chromatogram builder. It is important to note, each row represents a possible compound ID, however it is impossible to analyze when there are multiple peaks present in the “peak shape” column. The deconvolution process in the next step is critical to assign each ID row with a single peak.

*ADAP peak deconvolution:*

**Fig. 13:**
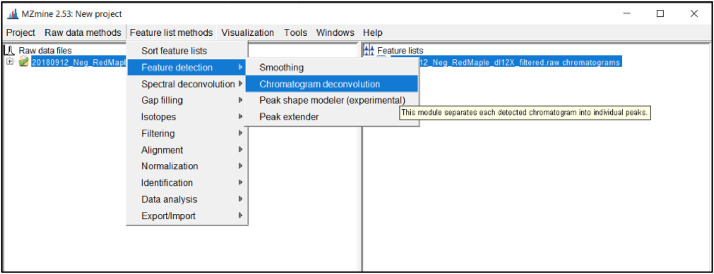
Screenshot to run ADAP peak deconvolution.

Next, the peaks from all chromatograms will be detected. Deconvolution is needed to separate the previously constructed chromatograms that span the entire duration. To run ADAP peak deconvolution select the chromatogram files on the left side of the screen and click *Peak list methods → peak detection → chromatogram deconvolution* as shown in Fig. 13.

**Fig. 14:**
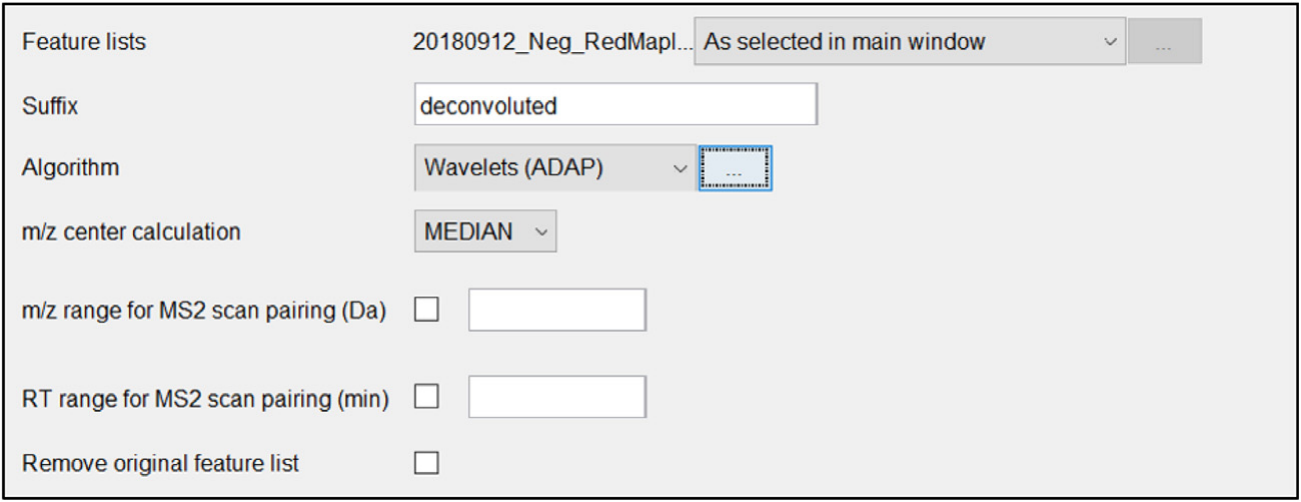
Parameter list for ADAP peak deconvolution.

This will open a window with parameters for the deconvolution step as shown in Fig. 14. Make sure the desired data lists are selected and add *deconvoluted* as the suffix. From the drop-down box select *Wavelets (ADAP).* The methodology used for this project follows the Wavelet ADAP process which is the most recent and comprehensive method developed [27].

**Fig. 15:**
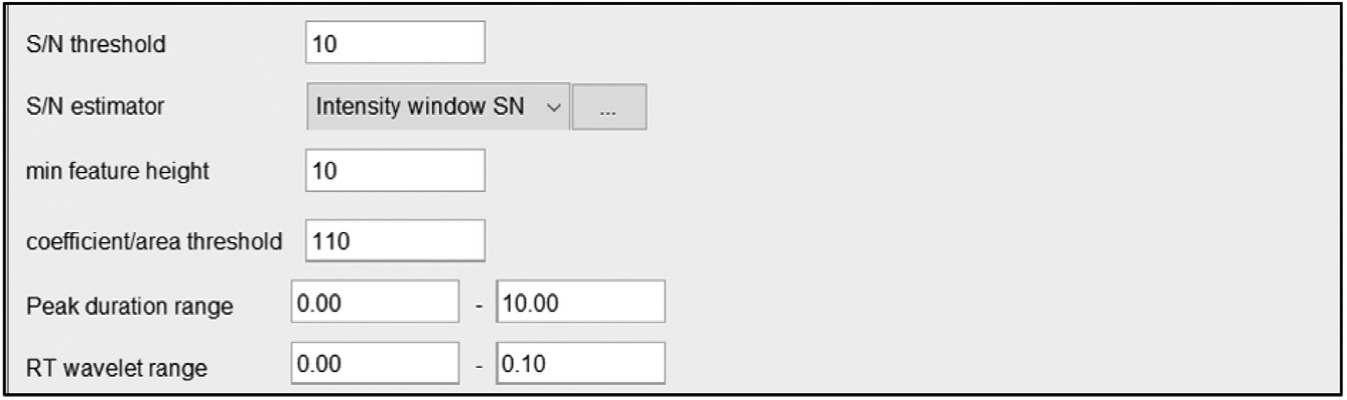
Algorithm parameter window.

After selecting Wavelets from the dropdown box, click on the ellipses to the right. This will open the algorithm parameter window shown in Fig. 15.

**Fig. 16:**
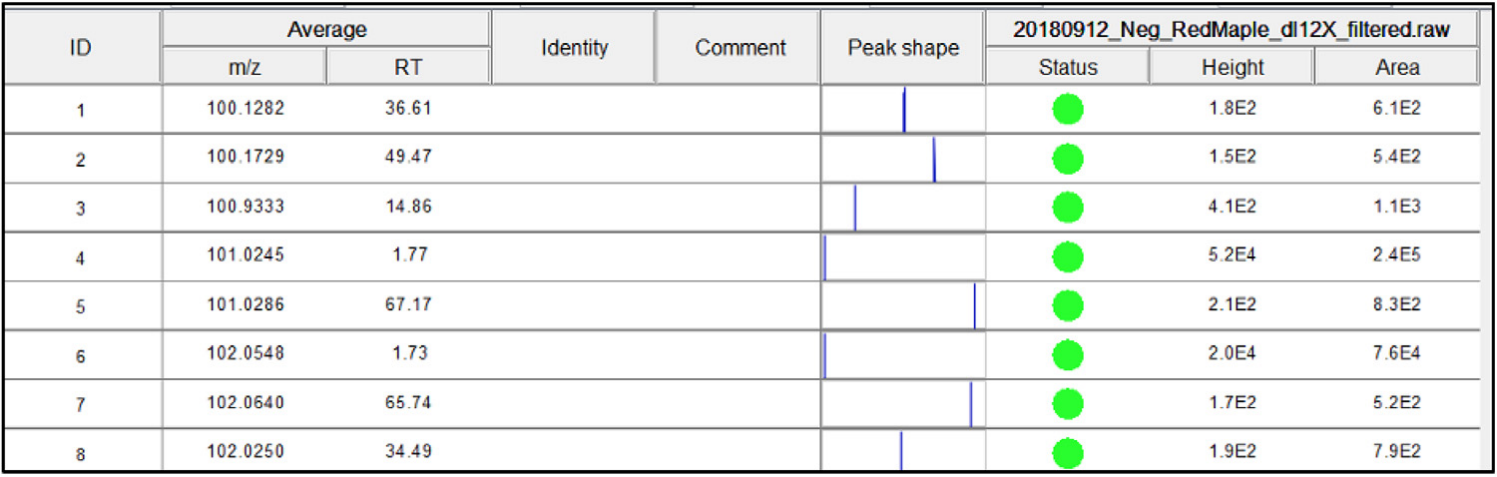
Example deconvoluted peaks of chromatogram.

Below are descriptions of each algorithm parameter shown in Fig. 16.

*S/N threshold:*

Signal to noise ratio threshold value greater than or equal to 7 will detect a small number of false peaks, this value compares peaks as 10x that of noise. 10 is a typical value to use for the Signal to noise threshold but can be adjusted based on results desired.

*S/N estimator:*

User can choose one of two estimators of the signal-to-noise ratio

1. The intensity window SN, which is tested on LC-MS datasets, utilizes peak height as signal level and standard deviation of intensities around the peak as the noise level;
  This is the Signal to Noise estimator used for this project because we are working with LC-MS data and not GC-MS.
2. Wavelet Coeff. SN, which is tested on the GC-MS datasets, utilizes continuous wavelet transform coefficients in order to estimate the signal and noise ratio. There is an analogous approach implemented in the R-package wmtsa [28,27].

*Min feature height:*

Minimum height of a feature. Should be the same, or similar to, the value - min start intensity- set in the chromatogram building. This value should be set to match the noise level and group intensity threshold used throughout running the program. Again, this value will change depending on the noise determined for a certain data set.

*Coefficient/Area threshold:*

This is a threshold for the maximum coefficient (inner product) divided by the area under the curve of the feature. Filters out bad peaks. This number must be chosen by looking at examples using the show preview button at the bottom of the window. This is the best coefficient found by taking the inner product of the wavelet at the best scale and the peak, and then dividing by the area under the peak. This value should also be similar to the RT Wavelet range set below. Somewhere around 1 or 2 should work for most data sets but can range up to 100.

*Peak duration range:*

Peak duration range is the range of acceptable peak lengths. Analyze chromatogram to determine how long peaks last. Auto entered at 0–10 adjusted down to 0.0–1.0 after analyzing the chromatogram, this will vary from dataset to dataset. The smaller the range the more confidence in the identified peaks.

*RT wavelet range:*

Upper and lower bounds of retention times to be used for setting the wavelet scales. Choose a range that is similar to the range of peak widths expected to be found from the data. Auto entered at 0-0.1, the auto entered range typically works well but can be increased to 1 or 2 when the data calls for it.

Once all algorithm parameters are set, click *OK* to return to the deconvolution parameter page. Then finish inputting those parameters shown below.

*m/z center calculation:*

Select “AUTO”

*m/z range for MS2 scan pairing (Da):*

Not entered/needed

*RT range for MS2 scan pairing (min):*

Not entered/needed

After the peak deconvolution program has run, a list of the chromatographic peaks will appear below the list of chromatograms in the *Peak lists* window on the right side of the screen. Fig. 16 shows an example of the deconvoluted peaks, this is updated from the detection step because peaks are now isolated and there is one clear peak for each row ID.

*Export data as csv file*

The data is now at the point where it can be exported to a .csv file so that it may be used in MFAssign R for formula assignment, identification, and interpretation. To export a csv, select the desired data and click *Feature List Methods → Export/Import→ Export .csv*

**Fig. 17:**
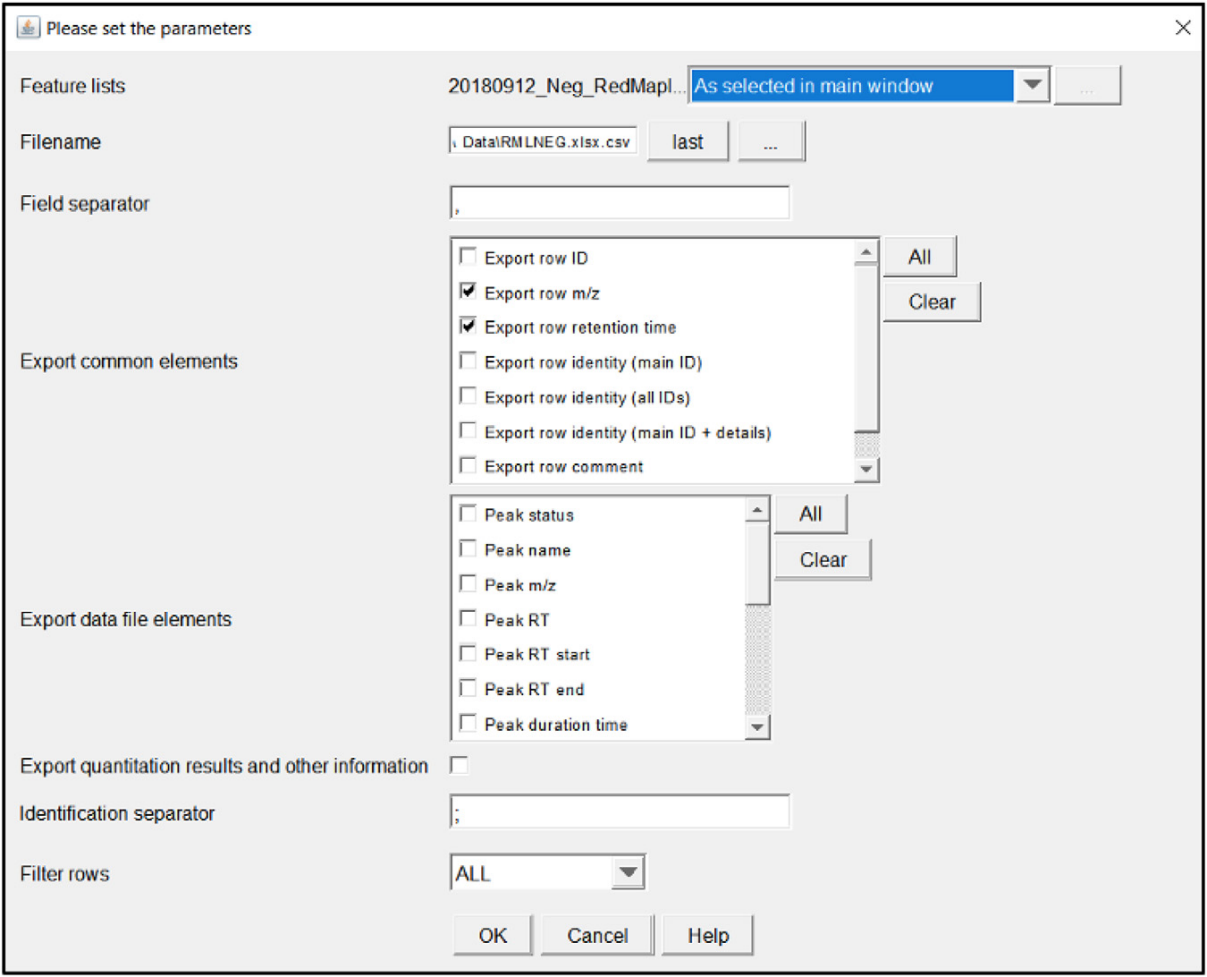
Export sample parameter page.

This will display a parameter setting screen shown in Fig. 17.

*Feature lists:* this is the same in all parameter windows, make sure the desired set of peaks/data is selected.

*Filename*: This is where the .csv will be stored; it is recommended to attach it to a blank excel sheet so it can be viewed and manipulated. It will still be stored as a .csv file. Make sure it is saved in a hard drive folder if using it in MFAssignR

*Field separator*: It will auto enter a comma in this field, change if needed.

*Export common elements:* The MFAssignR code requires row m/z, and optionally, row retention time data (useful for LC-MS analysis), select these items under the common elements section.

*Export data file elements:* The MFAssignR code also requires the Peak area data, select this item under the data file elements section.

*Export quantitation results and other information*: Unnecessary, leave this box unchecked.

*Filter rows*: Select “ALL”

*Preparing data for MFAssignR:*

Once the data from MZMine is successfully outputted into a .csv file,view the data in an Libre Office Calc [29] spreadsheet. Ensure the column order is as follows from left to right: row m/z, peak area, row retention time. This is the order needed for MFAssignR to process the data properly. Now ensure that the data is saved in a .csv file *not .ods or* .xlsx and save the data on a hard drive in a folder that has a defined pathway, this will be needed for the MFAssignR code.

### MFAssignR assignment

The next section utilizes MFAssignR [21]. To properly run this software the user will need to install both the programming interpreter for the R language [22] as well as the SDK “R Studio” [23], for both of which installation guides can be found online. This software uses multiple steps to assign formulas to the filtered mass spectrometry data created in the last step by MZMine. These steps, outlined in Fig. 1, consist of noise assessment, isotope identification, assignment, and recalibration. Blue text in this paper indicates direct code line from the MFAssignR package [21].

*Install packages:*

Installing packages is necessary to run the program. This only needs to be done once, can be commented out after they have been installed once. Lines can be “commented out” by inserting “#” in front of the desired line of code. A list of the packages needed for this program are listed below.

install.packages(“dplyr”)
install.packages(“tidyr”)
install.packages(“colorRamps”)
install.packages(“devtools”)
install.packages(“ggplot2”)
install.packages(“rmarkdown”)

The next step is to set the working directory, this will be the folder that the MFAssignR package was saved to on the user's computer. Will be different to what is shown below.

setwd (“C:/Users/name/Drive/MFAssignR-master”)
The next step is to write the call to install the MFAssignR program.
devtools::install(“MFAssignR”)

**Data Loading:**

Now loading the different necessary packages to do the formula assignment.

library(MFAssignR)
library(ggplot2)
library(dplyr)
library(tidyr)

Now set the working directory to where the .csv file produced from MZmine was stored and load the datafile. Must attach the .csv file produced from MZmine to the word “data” to be called on throughout the rest of the code.

setwd("C:/Users/lucyt/OneDrive/MFA Data")
Data <- read.csv("-ESI Final Compounds, Red Maple Leaf.csv")

Next step is to set the signal to noise ratio, this value allows the user to change the signal-to-noise ratio that will be multiplied by the estimated noise to determine the noise removal threshold. The SNRatio can be set from 0-10, but will likely be around 3. For the toxicity analysis project, the SNRatio was set to zero to ensure no legitimate peaks are mistaken for noise, since a majority of the noise was removed in the MZmine steps.

SNRatio <- 0
print("SNRatio")
0

*Signal to noise assessment:*

This is the signal to noise assessment section of the R markdown, demonstrating how to use the function KMDNoise()

Noise <-KMDNoise(Data, upper.y=0.3, lower.y=0.1)

This code shows how to extract the results of the KMDNoise() function so that they can be used

Noise [ ["KMD"]] #Plot showing the signal to noise estimation plot
KMDN <-Noise [ ["Noise"]] #Saving the estimated noise as a global variable in the environment
KMDN #Printing the noise so that it can be seen in the final report.
SNRatio = 0
SNplot(Data, cut = SNRatio * KMDN, mass = 319.1, window.x = 20, window.y = 10)

**Fig. 18:**
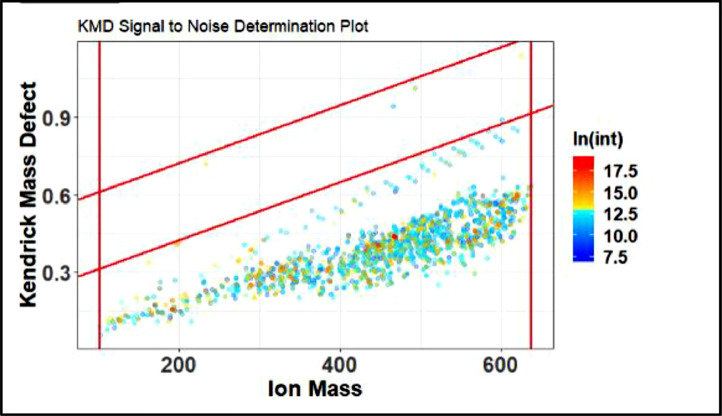
Improper noise determination plot.

This will produce 3 output screens that are attached below. Fig. 18 shows the KMD signal to noise determination plot. This plot is used just to look at the noise threshold relative to the mass peaks and their intensities. The goal here is to have most of the light to dark blue dots to be between the red lines whose location is specified by the code in line with (upper.y = 0.3, lower.y =0.1).

**Fig. 19:**
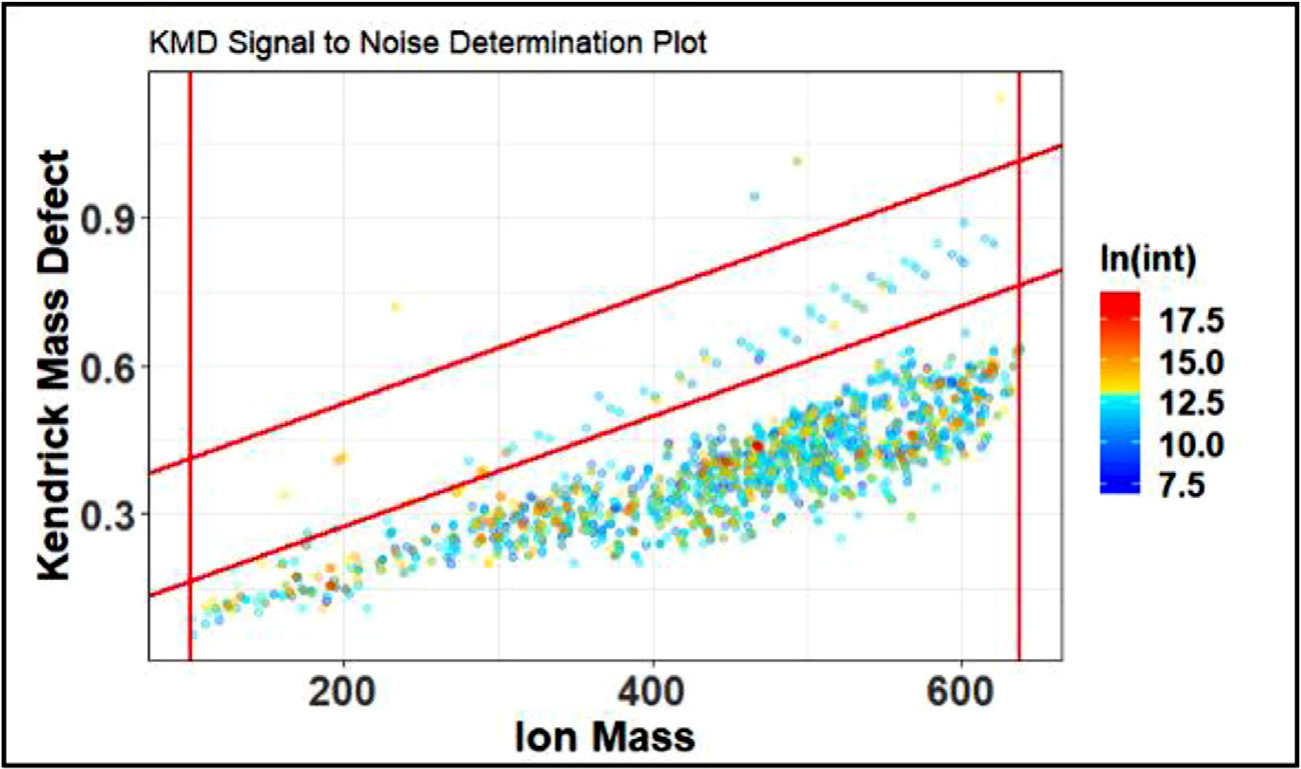
Proper noise determination plot.

After analyzing Fig. 18 it is clear the noise settings (red lines) do not contain the clearly separated blue points, this is remedied by adjusting the upper and lower y limits. After adjusting, a noise determination plot such as the one in Fig. 19 is produced.

The next figure window that will be produced from the Signal to Noise Assessment step is the KMDN value. This is a numerical representation of the estimated noise. This value will be saved in the global environment and used for future steps of the code.

**Fig. 20:**
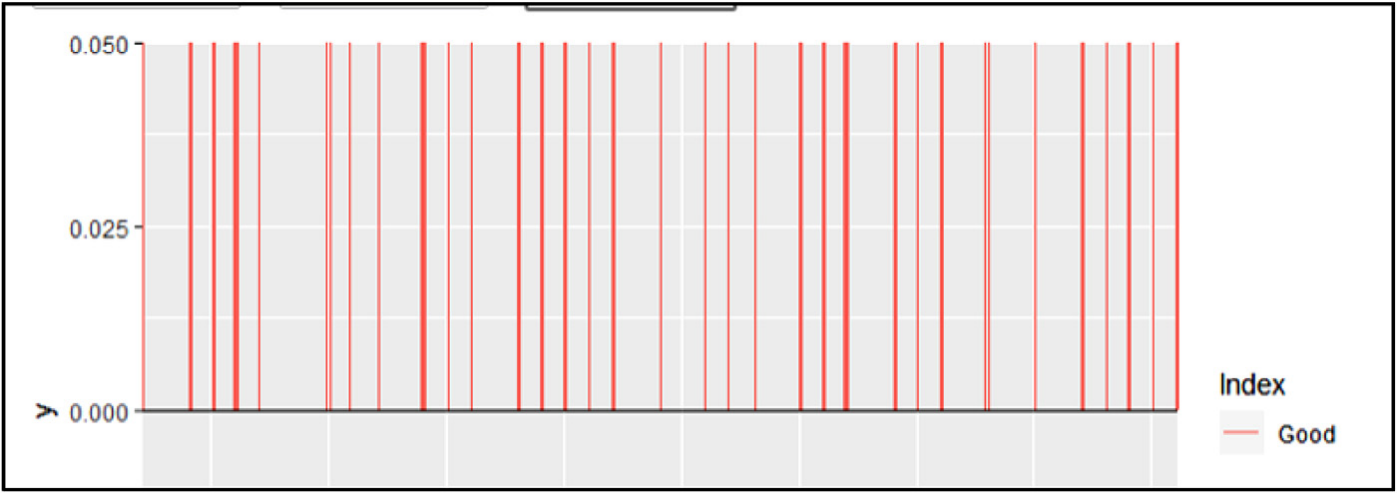
Noise index with SNRatio set to zero.

The last figure window that will be produced from this step is the noise index. This figure will change depending on what the SN Ratio is set to. If the SNRatio is set to 0, the visual will look like the one displayed in Fig. 20. There is nothing to analyze in the index if the SNRatio is set to zero since no peaks will be defined as “bad”. Most of the noise has already been removed in MZMine2 in this case so it is not necessary to have a non-zero SNRatio, but in other situations (predominately direct infusion) a ratio of 3–10 is generally appropriate.

**Fig. 21:**
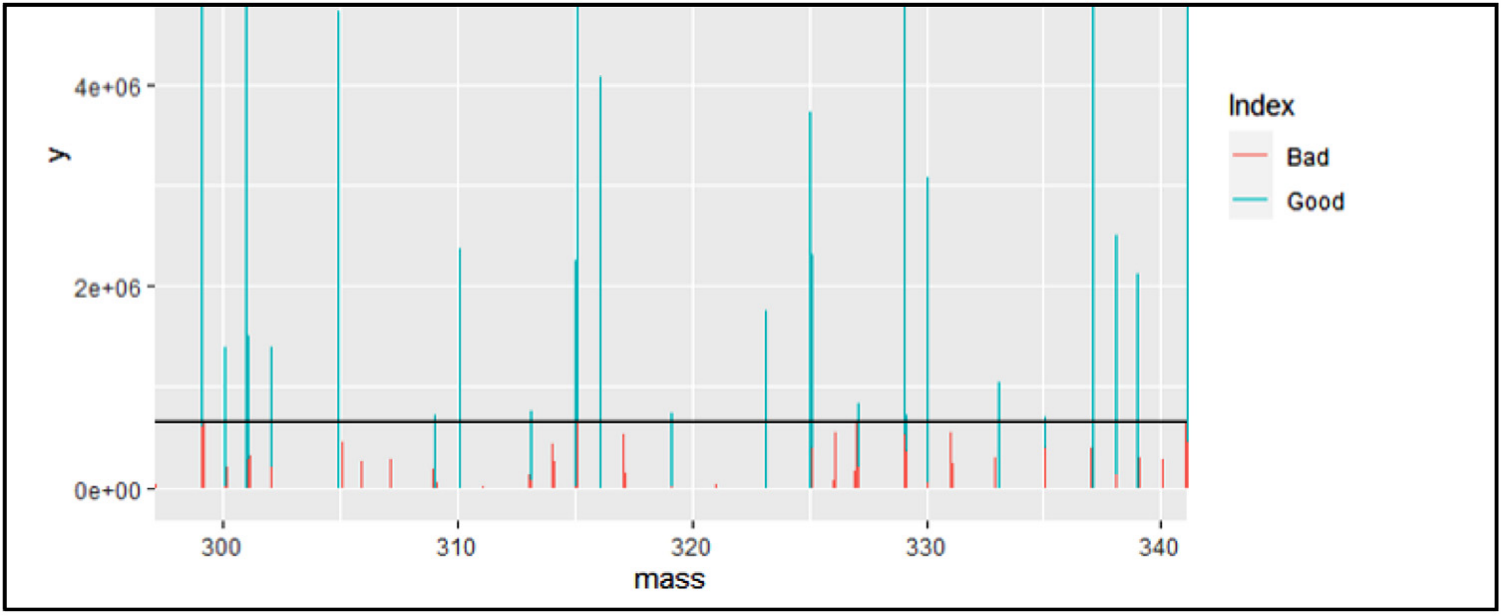
Noise index with SNRatio set to three.

However, if the SNRatio is set to 1–10, the noise index will look more like the visual in Fig. 21. This figure will display the blue peaks as “good” and the red ones as “bad”, the code will remove the peaks that are deemed “bad” from future steps of molecular formula assignments, so it is important to ensure these peaks truly represent noise. Ensure this by looking at the level of the “noisy” peaks, they should all be close to the same level and remember, more peaks that are removed can mean less confidence in the final toxin results.

*Isotope identification:*

**Fig. 22:**
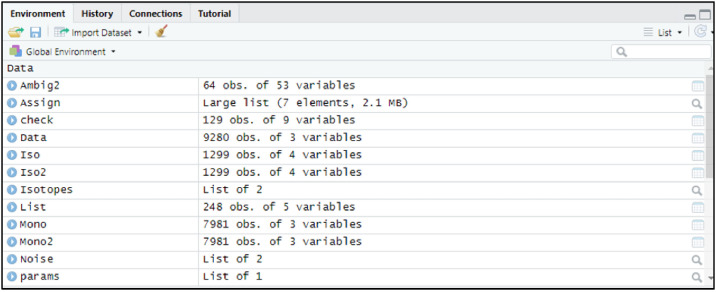
Screenshot of global environment in RStudio.

This section shows the usage of the IsoFiltR() function, which separates the single raw mass list into a list of likely monoisotopic masses ("Mono") and likely poly isotopic masses ("Iso"). The first line of code sets “Isotopes” as the results of the IsoFiltR function. Parameters for this include “Sulferr” and “Carberr”. This is the allowed error level when filtering sulfur and carbon. Typically, 3 is a good value for this but can be changed based on the mass accuracy of the instrument being used. The following two lines simply extract the two resultant mass lists from IsoFiltR and label them “Mono'' and “Iso” so that they can be used in later steps. There are no visual outputs for this section but extracted mass lists can be viewed in the upper right window/ the global environment as shown in Fig. 22.

Isotopes <- IsoFiltR(Data, SN = SNRatio * KMDN, Sulferr = 3, Carberr = 3)
Mono <- Isotopes [ ["Mono"]]
Iso <- Isotopes [ ["Iso"]]

*Prelim assignment:*

Next is the preliminary assignment step, this will assign molecular formulas to the initial peaks before any recalibration is performed. Be sure this section of code is updated based on what the target assignments are. The following line shows how to use the CHO only version of formula assignment. It is typically done to find molecular formula series to be used in recalibration. The parameters given in the code are standard for ESI negative mode. Table 1 shows standard parameters for both negative and positive modes.

Assign <- MFAssignCHO_RMD(Mono, Iso, ionMode = "neg", lowMW =50, highMW = 1000, ppm_err = 3, H_Cmin = 0.3, Omin = 1,
HetCut = "off", NMScut = "on", SN = SNRatio*KMDN)
Unambig1 <- Assign [ ["Unambig"]] #Unambiguous molecular formula assignments
Ambig1 <- Assign [ ["Ambig"]] #Ambiguous molecular formula assignments
Unassigned <- Assign [ ["None"]] #Unassigned masses
Plot1 <- Assign [ ["MSAssign"]] #Mass spectrum showing which peaks are assigned and
unassigned in the spectrum
Plot2 <- Assign [ ["Error"]] #Plot showing the error trend relative to mass for
assignments
Plot3 <- Assign [ ["MSgroups"]] #Mass spectrum showing the assigned molecular formulas
Plot4 <- Assign [ ["VK"]] #O/C vs H/C plot showing the assigned molecular formulas
Plot1
Plot2
Plot3
Plot4

**Table 1:** Preliminary assignment step parameters for negative and positive mode.

	Standard Parameters for Neg. Mode	Standard Parameters for Pos. Mode
ionMode	“neg”	“pos”
lowMW	50	50
highMW	1000	1000
ppm_err	3	3
H_Cmin	0.3	0.3
Omin	1	0
Mx	Not Included	1

“Mx” represents the sodium adduct that will be detected only in ESI Positive data and therefore is not necessary to include in the negative data processing. The following lines of code extract the outputs from the MFAssignCHO_RMD() function in the step above. This includes unambiguous and ambiguous molecular formula assignments as well as indexing the unassigned mass. The outputs will include 4 plots and 3 data frames.

The next lines of code are included to clear up some of the memory to keep the markdown running as fast as possible.

rm(Plot1)
rm(Plot2)
rm(Plot3)
rm(Plot4)
rm(Unassigned)
rm(Ambig1)
gc()

**Fig. 23:**
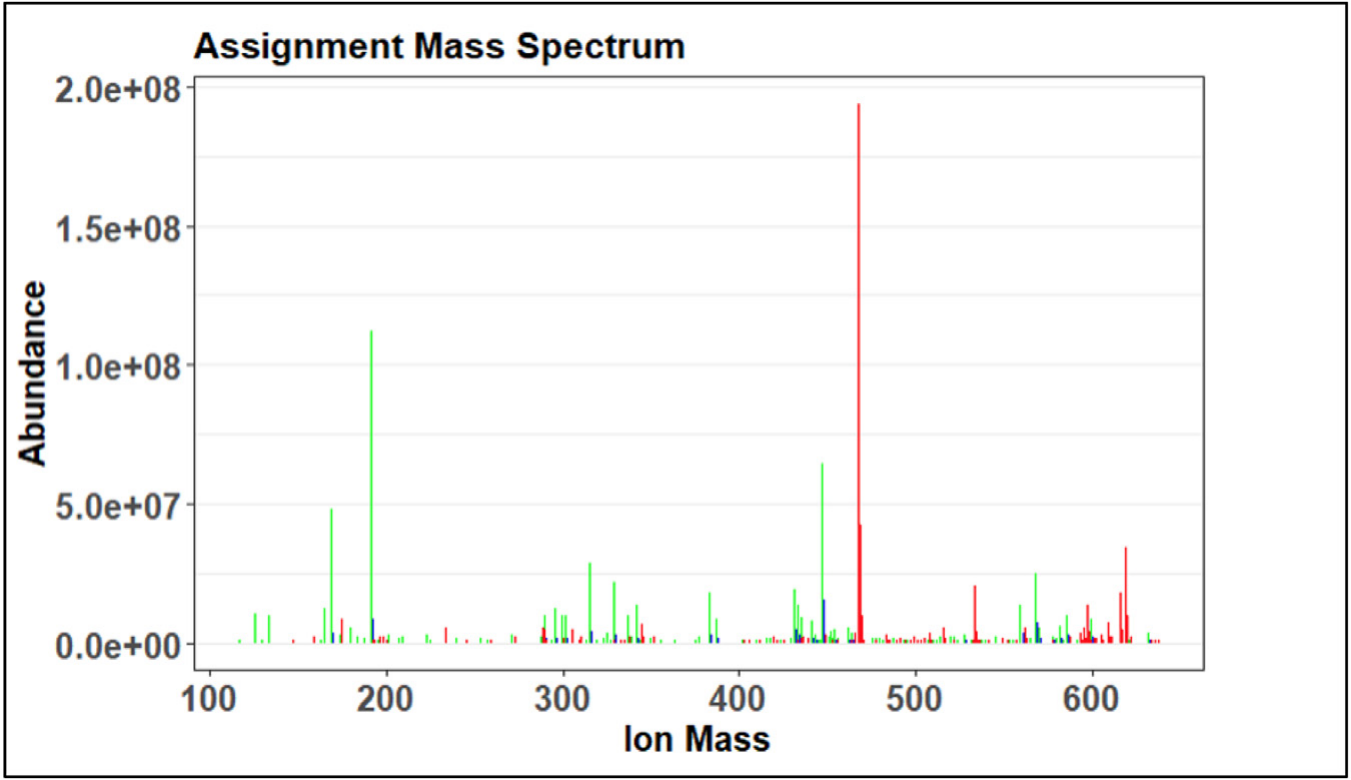
Example of an assignment mass spectrum.

The first visual produced by the preliminary assignment code is the assignment mass spectrum, an example is shown in Fig. 23. On this mass spectrum, red indicates an ambiguous or undefined assignment, green indicates an unambiguous or defined molecular formula assignment, and blue indicates a poly isotope. The goal of this visual would be to see as many green peaks as possible, however this is the preliminary step so it is expected to have more red peaks than will be in the final assignment step.

**Fig. 24:**
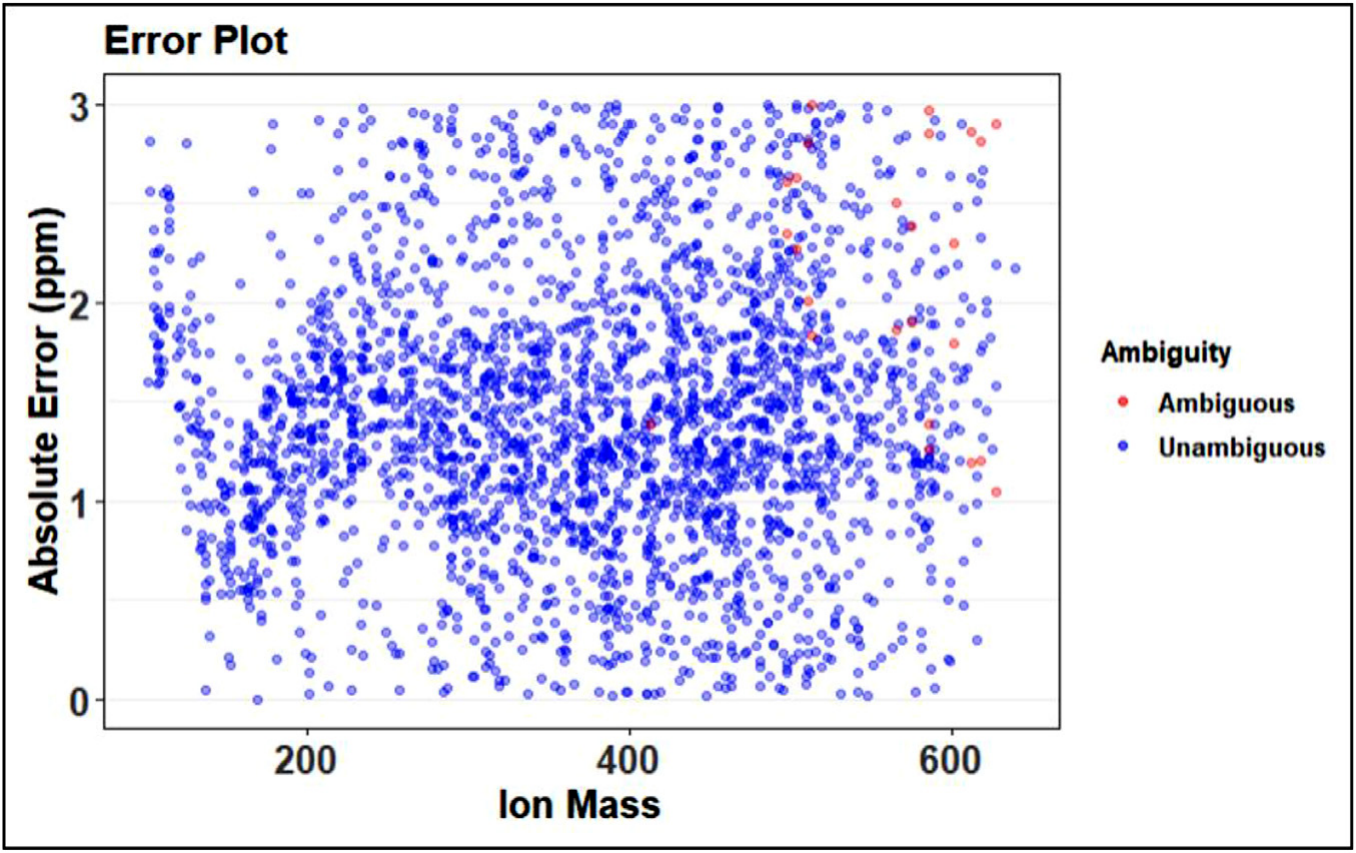
Example of error plot from preliminary assignment.

The next visual produced from this step of preliminary assignment is of the error plot, an example is shown in Fig. 24. The error should demonstrate some sort of trend that may vary depending on the instrument being used, for the preliminary error plot, trends are good. This example has a reasonable trend, though there are a couple point where the trend may be exceeding the error limits placed on the assignment. The error is raised by altering parameters within the KMDN step described above.

**Fig. 25:**
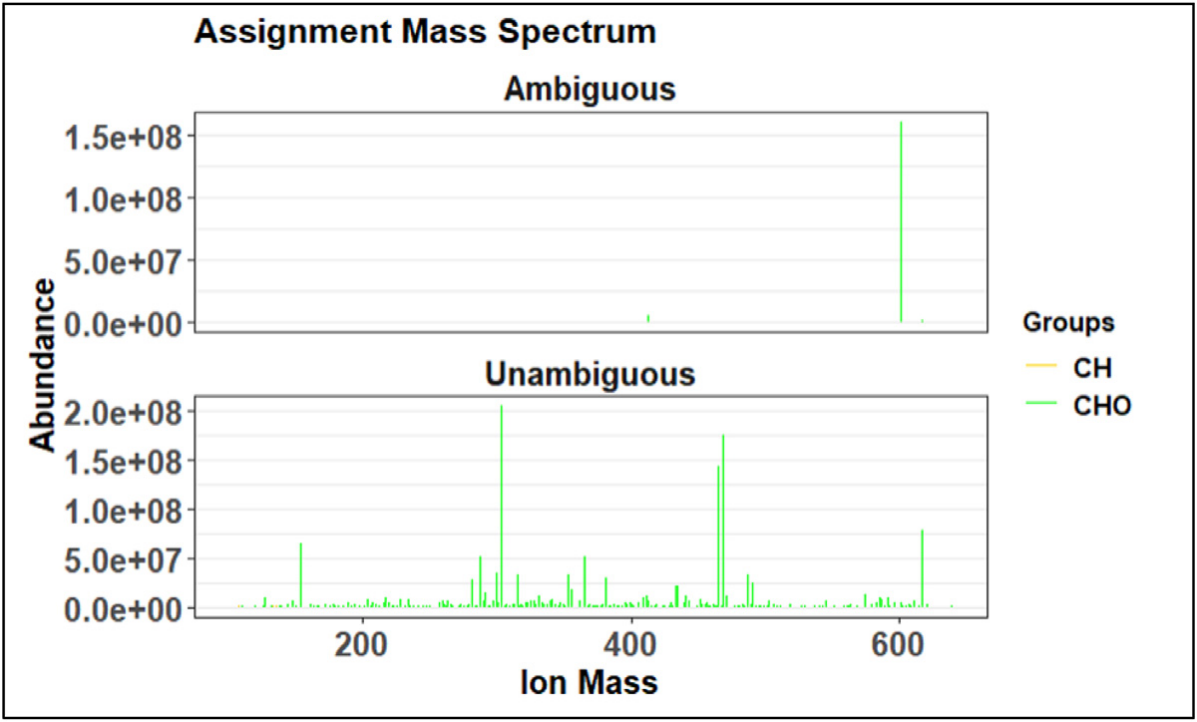
Example of the second view of the assignment mass spectrum from preliminary assignment.

**Fig. 26:**
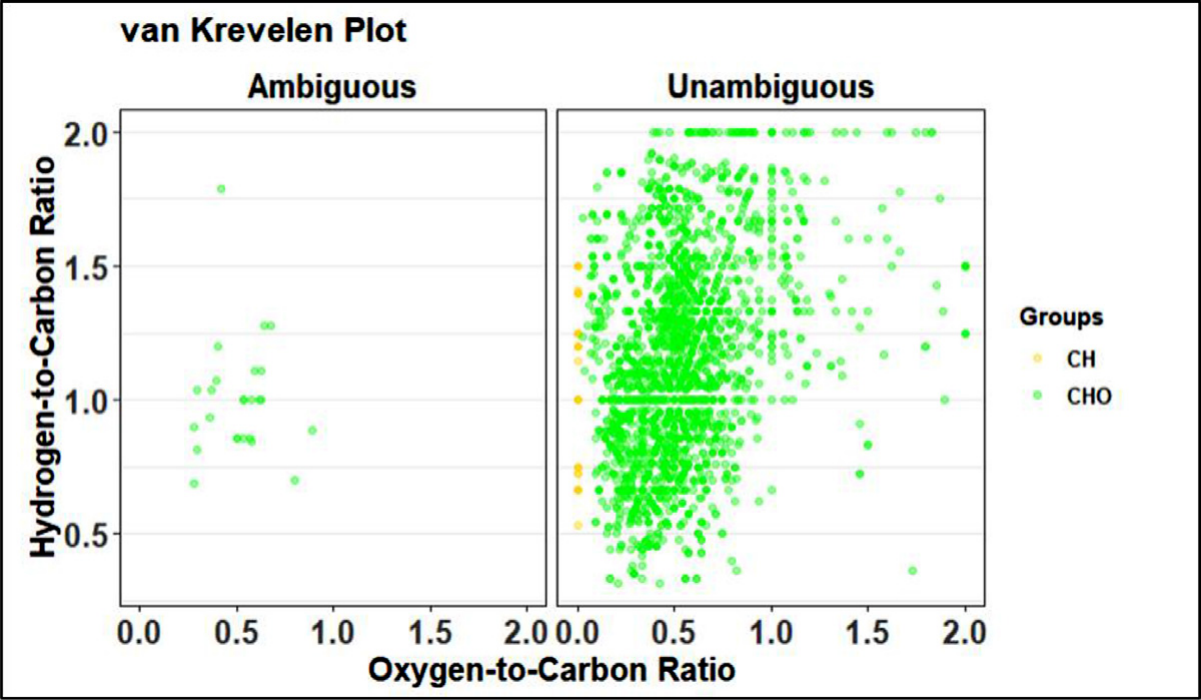
Example of a van Krevelen plot from preliminary assignment step.

The following two visuals shown in Figs. 25 and 26 are alternate views of the assignment mass spectrum and are useful to distinguish different species groups such as CH, CHO, CHNO, etc. When more chemicals are added to the assignment code, these figures can determine if the added chemicals are causing more peaks to be defined as ambiguous.

*Recalibration*

The recalibration step requires the most user input. If available, representative samples should be run first to determine the set function “recalibrants” with confidence. The first two lines of code before the “check” step are optional but are useful for setting up recalibration in LC-MS runs because they remove duplicate mass/formula combinations (from isomers) that cause issues in recalibration calculations. Typically, these lines are set up to retain the most abundant example of each mass/formula combination. The goal of this is to remove all series that may have a Series Score less than 1, because a Series Score less than one causes issues for recalibration. This filtering step only impacts the selection of recalibrant series and the calculation of recalibration correction terms, all masses will be recalibrated in the function. This makes it easier to select formulas that cover the entire mass range spectrum.

```{r, echo = FALSE, message = FALSE, warning = FALSE}
Unambig1 <- Unambig1 [order(-Unambig1$abundance),]
Unambig1<- Unambig1%>%distinct(formula, .keep_all=TRUE)
check <- RecalList(Unambig1)

**Fig. 27:**
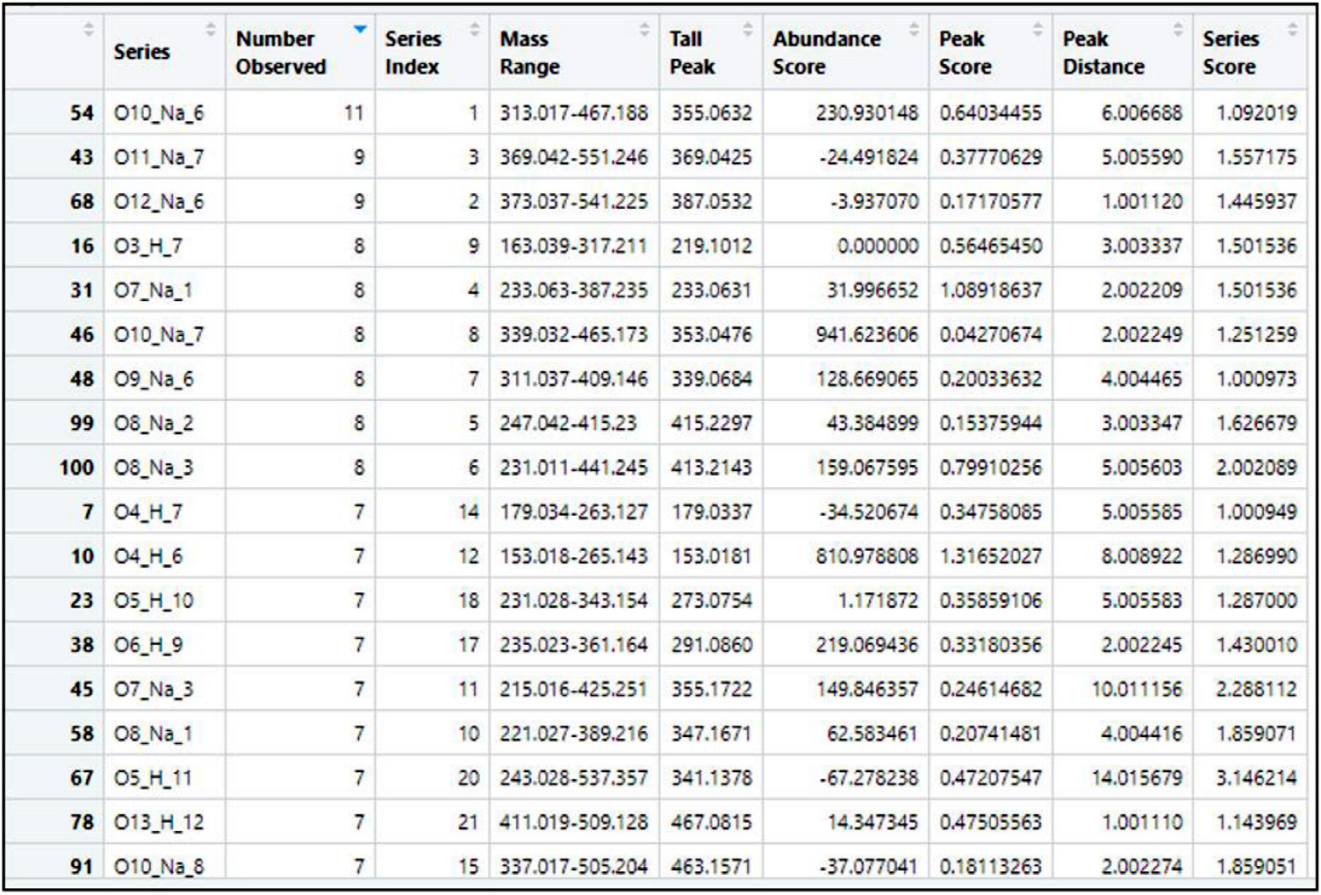
View of the “check” table within the recalibration step of code.

After running the above section of code, the user must run a qualitative check of “recalibrant” series and mass recalibration. This is done by examining the “check” page that can be found in the upper right-hand global workspace, also found in Fig. 22. After selecting the check, the table comes up in the upper right-hand window as shown in Fig. 27.

**Fig. 28:**
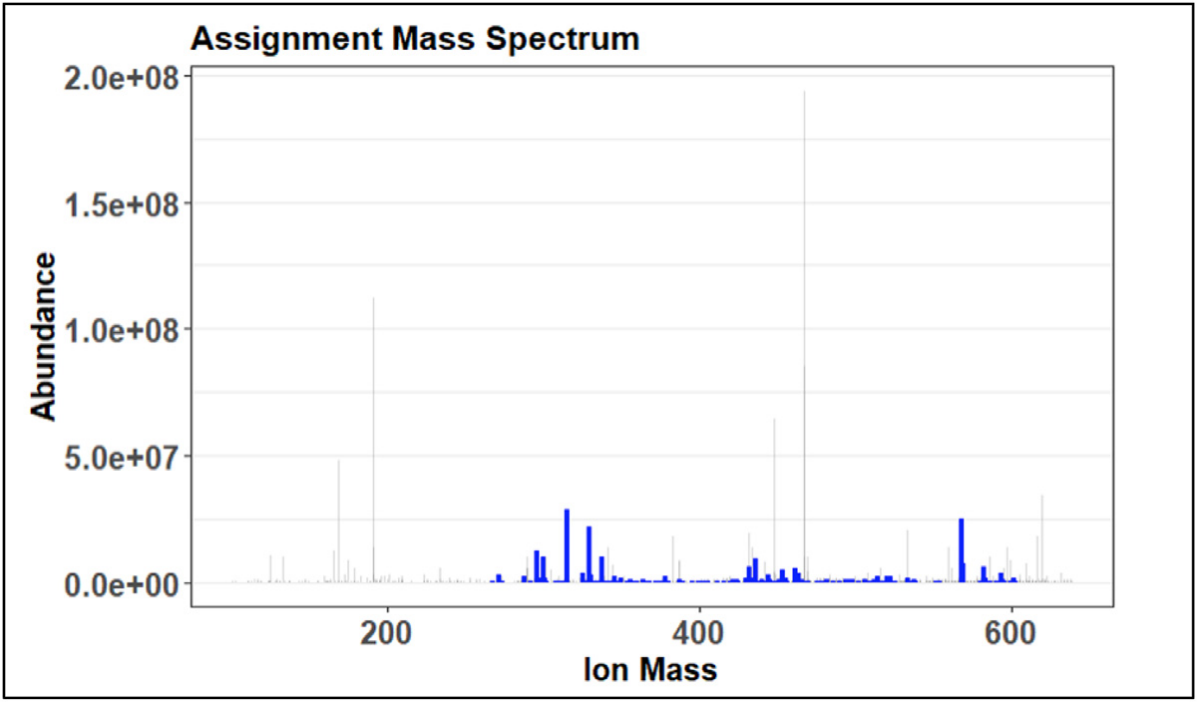
Sample Assignment mass spectrum showing range of ion mass assignment for the recalibration step.

This is the data that will be analyzed to determine the series used to create the recalibrated mass spectrum. The overall goal is to get the longest continuous spectrum of ion masses covered by the series selected. This can be visually checked in Fig. 28, the selected spectrum is indicated by the blue color.

Selecting series is determined on a set of parameters found in the column section of the “check” table.

Examine the “check” table, in the environment to select 5 or 6 different chemicals to use in full assignment step. When selecting formulas, want mass ranges that cover a large range of the total mass spectrum (need to know highest and lowest mass range) while also examining the following parameters outlined in Table 2.

**Table 2:** Parameters for selecting series in recalibration.

Parameter Name	Ideal Value	Notes
Number Observed	Higher	*sort rows highest to lowest
Mass Range	Largest	*if able, select larger mass ranges instead of smaller
Abundance Score	Positive	*optimally positive
Peak Score	Close to 0	*some room (can be above or below)
Peak Distance	Close to 1	*Least Important, can be closer to 2
Series Score	Close to 1	*Critical, do not go below 1

**Fig. 29:**
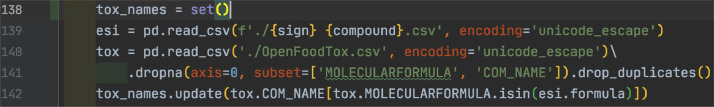
View of the “check” table within the recalibration step of code.

When formulas are selected, insert them in the following code of the Recal function. Depending on confidence of formulas selected and peak and series scores, the mzRange is typically around 15 but can be increased if selected series are not completing the necessary range coverage. After the following lines of code are run the plot in Fig. 29 will be produced and analyzed, in some cases the selected series will not provide a strong enough range and the code will error. In this case new series must be selected.

Test <- Recal(Unambig1, peaks = Mono, isopeaks = Iso, mzRange = 15, mode = "neg", SN = SNRatio*KMDN, series1 = "O7_H_6", series2 = "O9_H_5", series3 = "O6_H_7", series4 = "O9_H_10", series5 = "O9_H_12", series6 = "O12_H_11", series7 = "O5_H_5")
Plot <- Test [ ["Plot"]]
Plot #This plot can take time to generate
Mono2 <- Test [ ["Mono"]] #Recalibrated monoisotopic mass list
Iso2 <- Test [ ["Iso"]] #Recalibrated isotopic mass lists
List <- Test [ ["RecalList"]] #The list of formulas/masses used as recalibrants
print("mzRange")
15

*Full assignment*

It is important that this section of code is updated with all necessary elements depending on assignment goals.

Use of MFAssign_RMD() for molecular formula assignment. The notable differences between the full and preliminary assignment steps are the option to add chemicals (Sx, Nx, Px, Clx, etc.) to the assignment process, and the isotope error (set to 3), and having Sulfur check and MSMS set to “on”. Following the code below, besides what was indicated in Table 1, everything remains the same in negative and positive modes.

Assign <- MFAssign_RMD(Mono2, Iso2, ionMode = "neg", lowMW =50, highMW = 1000, Sx = 1, Nx = 3, ppm_err = 1, iso_err = 1, H_Cmin = 0.3, Omin = 1, SulfCheck = "on",
HetCut = "off", NMScut = "on", SN = SNRatio*KMDN, MSMS = "on")

It is important to note that the addition of more elements to formula assignment beyond C, H, and O can lead to increased ambiguity in the formula assignments due to more chemically feasible combinations of elements being possible, particularly at high masses [34]. MFAssign has been written to include data-dependent pathways to decrease the ambiguity in formula assignments using formula extension “spiderwebs”, which decreases the likelihood of ambiguous assignments, but care should be taken when including multiple non-oxygen heteroatoms to be confident in the formula assignments. To mitigate this response, the user can run the same data set through the code more than once, adding different chemicals (of increasing error) to the full assignment step each time. It is recommended to run through the data once with only CHO and no user added chemicals, this should result in no ambiguous assignments and offers a solid baseline of confirmed formulas that can be compared to the formulas produced when more chemicals are added. A good work progress for this method is to run through CHO first, then add nitrogen for the CHNO assignment, then add Sulfur and chlorine. When checking chlorine assignments, check for the isotope Cl37 and confirm there is a good peak at the correct retention time.

Once as many formulas as possible are assigned and verified, the data can be run through once more with the addition of phosphorus. Phosphorus is difficult to assign because there are no isotopes, so it can swing the formula assignment results. When analyzing phosphorus assignments some unreasonable groups such as PNO2 will be present, these can be excluded from the confirmed formula assignment. This is because of the lack of enough oxygen to support what should likely be a phosphate group given the presence of phosphorus. This “staging” method may not be necessary for all data sets, some will produce satisfactory results with all chemicals included in the first run. Overall, when the output formula assignments are reviewed if any seem unreasonable they can be confirmed by comparing the mass in the data with the theoretical “exact mass” of that compound with an error range of around 3 ppm. The key is to always be critical of formula assignments from a chemical feasibility angle based on knowledge of the sample and analytical method, particularly when useres are first getting started.

Extraction of data from the MFAssign_RMD() function. It has the same format as the MFAssign CHO_RMD() function. The code for outputting the final visuals and molecular formula assignments is below:

Unambig2 <- Assign [ ["Unambig"]]
Ambig2 <- Assign [ ["Ambig"]]
Unassigned2 <- Assign [ ["None"]]
Plot1 <- Assign [ ["MSAssign"]]
Plot2 <- Assign [ ["Error"]]
Plot3 <- Assign [ ["MSgroups"]]
Plot4 <- Assign [ ["VK"]]
Plot1
Plot2
Plot3
Plot4

Plots 1 through 4 match the ones produced in the preliminary assignment step shown in Figs. 24–27. Progress made in the recalibration step can be seen by comparing two of the same plots in prelim and full assignment steps. Changes that are expected are less peaks in the ambiguous graphs and more in the unambiguous. The error plot should have no trend and the van Krevelen plot should be defined.

Final step is to write the output csv code. This can be done by the following line of code for the desired data. Typically, the final results will be stored under the name “Unambig2” if the results call for it or if the user would like to analyze the ambiguous assignments as well, “Ambig2” can also be outputted as long as “Ambig=On” is specified in the MFAssign_RMD section of code. These object names can be changed by the user as desired.

write.csv(Unambig2, "MFARedMapleLeafprelim.csv")

### ToxAssign filtering process

ToxAssign [26] is a tool developed by that utilizes python data mining tools, the NIH's PubChem API tool [30], and the Open Food Tox Database (OpenFoodTox) published by European Food Safety Authority [31,32]., The European Food Safety Authority's scientists have produced risk assessments for more than 4950 substances. Each substance, which has been evaluated has a summary of its effect on human health. In addition, based on the relevant legislation and intended uses, there can also be animal health and ecological hazard assessments provided as well. All of this information is collected and structured into the European Food Safety Authority's chemical hazards database: OpenFoodTox. OpenFoodTox is fully open source data meant for substance characterization. For researchers it also provides links to other output including background European legislation. Finally, OpenFoodTox provides a summary of the available critical toxicological endpoints and any reference values.

ToxAssign is broken into five modules, one module that automates execution and the other four that perform specific operations. The automation tool creates a directory for each sample, calls the PubChem module, toxic filter module, match module, the merge module three times, then the toxic filter module again. The PubChem module first matches potential toxic formulas from the list of data from MFAssignR against the local toxic table, then retrieves the full records from PubChem, then sorts them based on chosen records and writes them to files. The toxic filter module takes the toxic records and filters the records by acute toxicity and writes them to file. The match module matches any records not found on PubChem against a local table of records and writes the found records and still unfound records to file. Finally, the merge module takes the files from each sample and merges them together then writes them to file.

To install this tool, the user will need to go to the Github page for this project [26], where the instructions for installation, operation, and output are detailed. As well the user may need to download and install the python language [25], for which guides can be found in the reference below. To tell whether it is needed or not, open a terminal and type “python3”, if there is no error then python is already installed. Otherwise, go to the reference below, navigate to the downloads page, and download the proper install for the computer operating system the operator is using.

*PubChem:*

As is demonstrated in Fig. 1, the first module to be called is the PubChem module. This module first matches the formulae given out by MFAssignR and matches them against a local table of potentially toxic compounds as shown in Fig. 29.

**Fig. 30:**
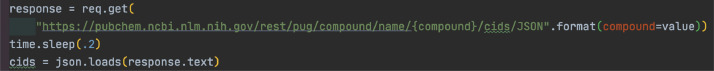
View of the “check” table within the recalibration step of code.

**Fig. 31:**
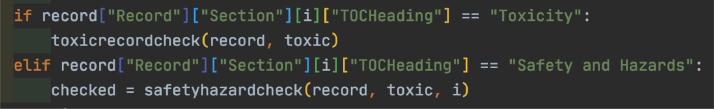
View of the “check” table within the recalibration step of code.

Then, in the same way as Fig. 31, the module queries PubChem with the compound name to get the CID or list of CIDs that match that compound. Next, as demonstrated in Fig. 30, the module uses these retrieved CIDs to get the full records for each compound from the PubChem database, waiting a fifth of a second between each to not overload PubChem's servers.

Next the module sorts the records by different fields that may or may not show up. To do so, the module loops through the fields of the record looking for either ones relating to toxicity or to food safety, checking in such a way as is shown in Fig. 31. When it finds one of these fields it then writes it to a data object that then gets written to file.

*Toxic filter:*

**Fig. 32:**
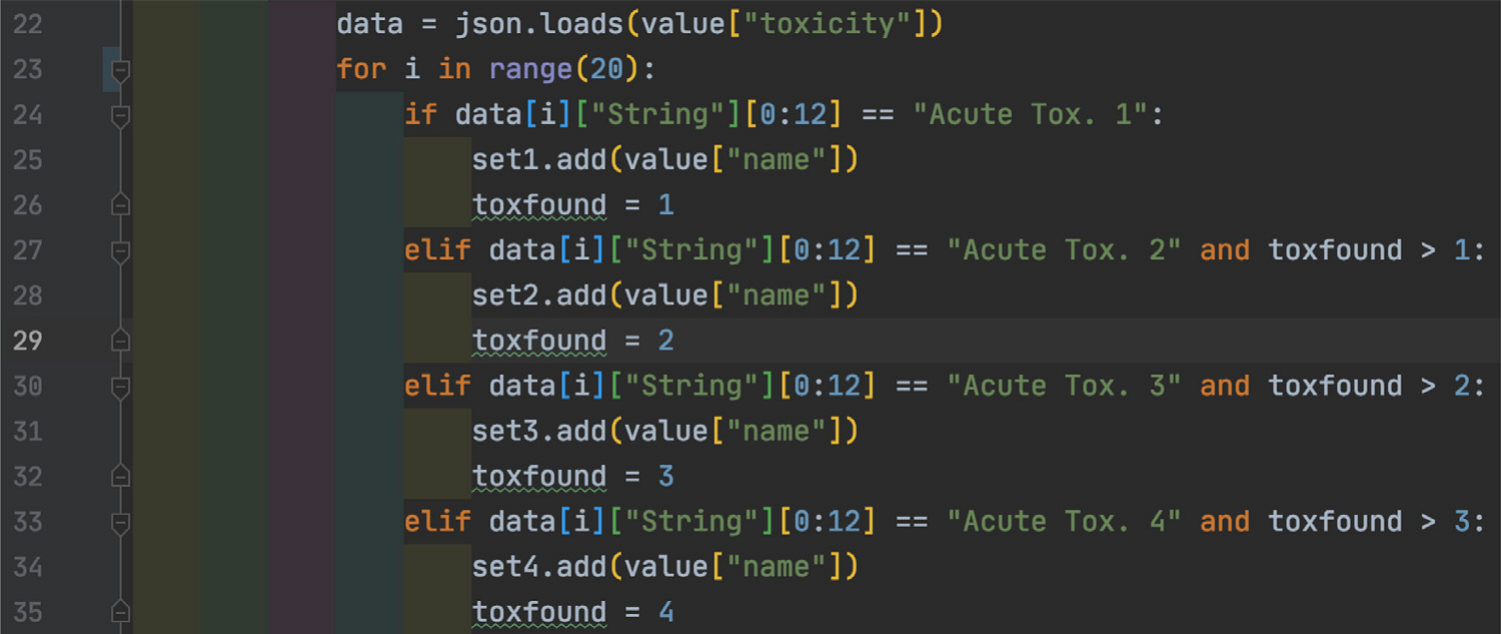
View of the “check” table within the recalibration step of code.

The next module to be ran by the automation, as shown in Fig. 1, is the toxic filter. This module takes the toxic record output by the PubChem module and filters it into five categories then writes them to a file to make understanding the data much easier. This is done by looping through each classification of the record and matching it against different acute toxic levels as in Fig. 32. The highest matching toxicity is then stored to a data object and finally written to a file.

*Match:*

**Fig. 33:**
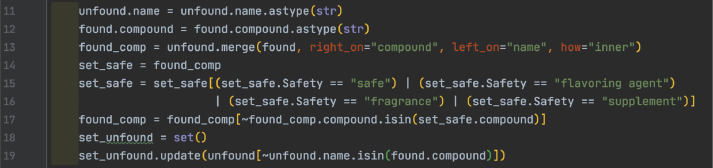
View of the “check” table within the recalibration step of code.

Next, the match module matches all records that were not found in the PubChem database against a local table of previously unfound records. Any that are matched are outputted to a file along with their safety class and any that are not are put back into the unfound file to be added to the local table (Fig. 33).

*Merge:*

**Fig. 34:**
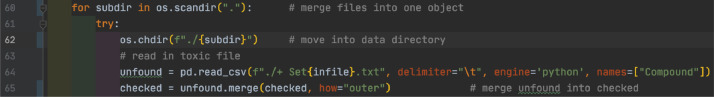
View of the “check” table within the recalibration step of code.

The final module is the merge module, which takes the files from individual samples and merges them together for help in decisions about further analysis of the set, as well as easier analysis for repeated analysis of the same sample. This is done first as shown in Fig. 34 by looping over all of the directories and first moving into them, then merging the positive data set into a data object within the code.

**Fig. 35:**
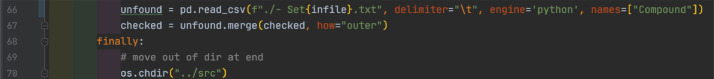
View of the “check” table within the recalibration step of code.

Next, the module combines the negative data into the same object and finally moves out of the directory to begin again. As demonstrated in Fig. 35, this uses the pandas utility's merge function, which combines two sets of data that match along a given key. Because there is only one column in both datasets the key does not need to be specified.

At the end the data object is written to an output file with the shared name of the in file as its name. This operation is performed three times: once for the toxic data, once for the unfound data, and once for the unchecked data. These are all merged to help choices for which standards to use to sample the entire data set, to help with adding to the remove data file, and to help with checking records that did not have any fields in the PubChem database.

*Toxic filter:*

As is shown in Fig. 1, the toxic format module is run one more time, and this is to format the merged toxic file to make it easier to understand, again for the use of picking standards for further analysis through comparing the sample to chosen standards.

### Validation against Compound Discoverer method

A goal of this paper was to replicate a procedure performed previously using an expensive and hard to obtain piece of software, Compound Discoverer, in an open source and easy to access and use format. This is to make the process not only less expensive but also more automated and easier to use, and thus, more accessible to under-resourced labs and more attractive to researchers to build upon this method in the future. To that end a part of this paper will be devoted to comparing this analysis to the previous analysis done using Compound Discoverer.

**Fig. 36:**
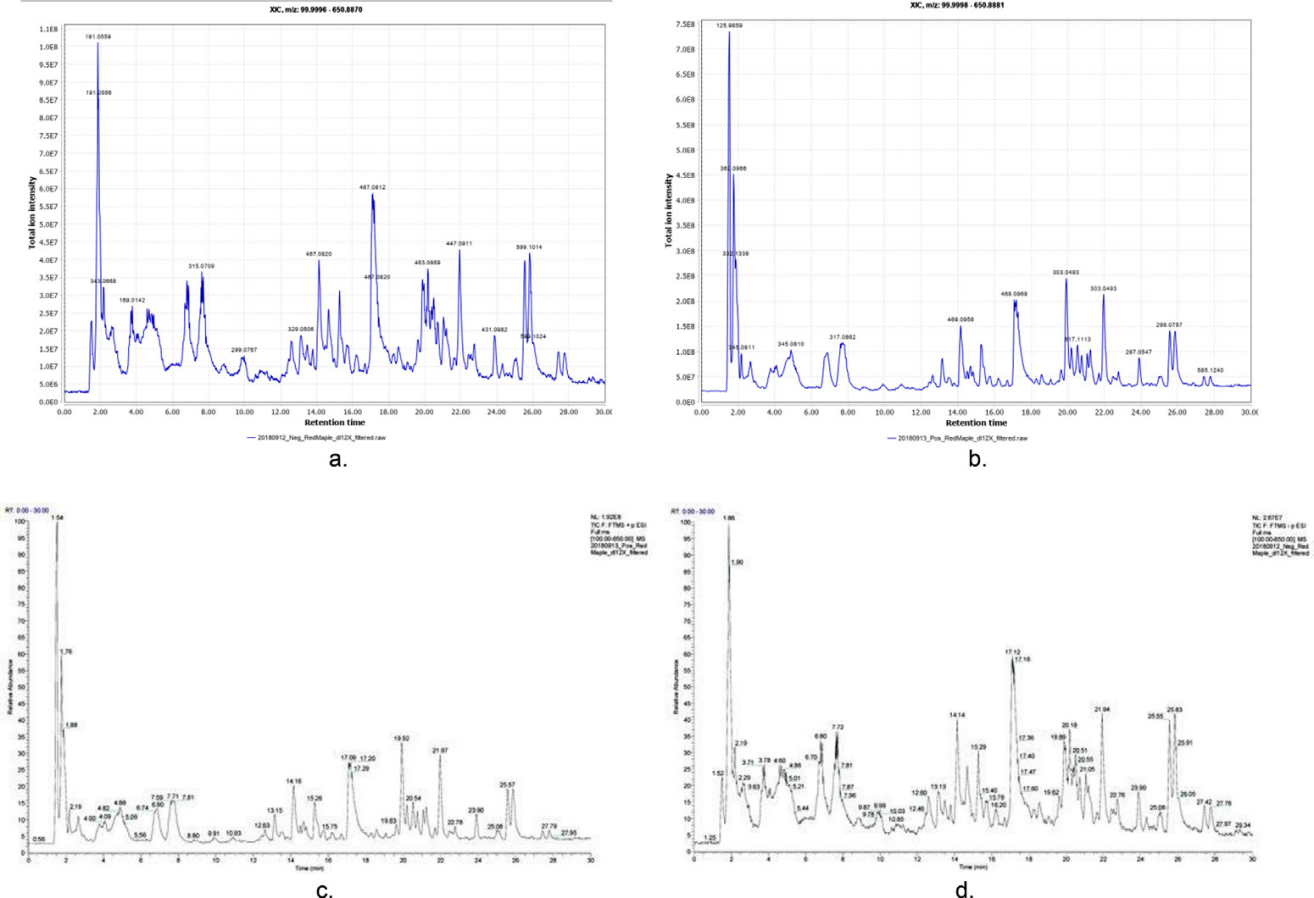
Total Ion chromatogram of current process (a,b) and previous process (c,d) [Pearce].

The findings of this paper in regard to the volume of toxic compounds, while larger, are also still lacking one step of validation in their analysis. Also, it should be pointed out to this end this process finds only toxic compounds in the sample, while Compound Discoverer works to find all compounds within the sample, toxic or not. This process also uses different data than Compound Discoverer and different validation methods, so comparison becomes difficult. That said, looking at the comparison between the total ion intensity of the new data and the relative abundance of the old data shows a similar set of data (Fig. 36).

Looking at Table 3, the new process identifies all of the toxic elements the previous process identified with more detailed information than the previous process was able to provide automatically. Coincidentally, none of these are ranked as having an acute oral toxicity as higher than level 4, while some of them are irritants to skin and eyes. While all of the potential toxic elements were identified, none of the non-potentially toxic elements were identified as this new process only matches formulas to the OpenFoodTox database. This can present a limitation, as the compound must be listed in the given toxic compound database to be recorded, while in the previous method compounds that are not already known to be toxic can be identified. This method is more useful for identifying exactly in what way and amount a compound is toxic much more rapidly, however, because of the extra information given about each compound in the output. It should be noted that these are tentative matches as there is no structural information for these formula assignments included in the current comparison.

**Table 3:** Toxic elements identified by new process.

Previous analysis [Pearce]	New analysis	New classification
3-methoxybenzaldehyde	3-methoxybenzaldehyde	Skin and eye irritant 2
4-methoxybenzaldehyde	4-methoxybenzaldehyde	Acute toxic 4
coumarin	coumarin	Acute toxic 4
L-glutamic acid	glutamic acid	Potentially toxic
L-phenylalanine	phenylalanine	Eye irritant 2
citric acid	citric acid	Skin and eye irritant 2
L-aspartic acid	aspartic acid	Potentially toxic
naringin	naringin	Skin and eye irritant 2

## Future work

This method still needs further validation and automation to overcome the gaps in the PubChem database as well as differences in naming conventions between the European Food Safety Authority ’s database and the NIH's database. For now, this is resolved with a manual search of records that do not match and a local storage of those records in a csv. Future extensions to this free software could work to use other services and online databases to match the European Food Safety Authority ’s recorded name to the NIH's recorded name and store the record in local JSON storage. This could also be used to store information about the compounds that have already been searched, allowing for faster processing and less strain on PubChem. Another potential upgrade includes building in functionality to analyze fracturing runs and retention time of compounds against standards using the MZCloud database [33] to automate the verification and certification of compounds to make the process fully automated. Finally, although the purpose of this free and open source software toolchain was to identify potential toxic substances in potential alternative or resilient foods [35,36], however the approach has a much larger potential set of applications including identifying toxicity in botanical substances that could be used for food or additives, feed for animals or for cosmetic ingredients.

## Declaration of Competing Interest

The authors declare that they have no known competing financial interests or personal relationships that could have appeared to influence the work reported in this paper.
